# Quantum Spin Exchange Interactions to Accelerate the Redox Kinetics in Li–S Batteries

**DOI:** 10.1007/s40820-023-01319-8

**Published:** 2024-01-29

**Authors:** Yu Du, Weijie Chen, Yu Wang, Yue Yu, Kai Guo, Gan Qu, Jianan Zhang

**Affiliations:** Key Laboratory of Advanced Energy Catalytic and Functional Materials Preparation of Zhengzhou City, College of Materials Science and Engineering, Zhengzhou University, Zhengzhou, 450001 People’s Republic of China

**Keywords:** Metal phthalocyanines, Spin polarization, Electrocatalysis, Li–S batteries

## Abstract

Spin-engineering with electrocatalysts have been exploited to suppress the “shuttle effect” in Li–S batteries. Spin selection, spin-dependent electron mobility and spin potentials in activation barriers can be optimized as quantum spin exchange interactions leading to a significant reduction of the electronic repulsions in the orbitals of catalysts. Herein, we anchor the MgPc molecules on fluorinated carbon nanotubes (MgPc@FCNT), which exhibits the single active Mg sites with axial displacement. According to the density functional theory calculations, the electronic spin polarization in MgPc@FCNT not only increases the adsorption energy toward LiPSs intermediates but also facilitates the tunneling process of electron in Li–S batteries. As a result, the MgPc@FCNT provides an initial capacity of 6.1 mAh cm^−2^ even when the high sulfur loading is 4.5 mg cm^−2^, and still maintains 5.1 mAh cm^−2^ after 100 cycles. This work provides a new perspective to extend the main group single-atom catalysts enabling high-performance Li–S batteries.

**Supplementary Information:**

The online version contains supplementary material available at 10.1007/s40820-023-01319-8.

## Highlights

- Compared with the traditional single-atom catalysts (SACs), the Mg SACs with axial displacement is accurately fabricated on the functional carbon nanotubes.
- The electronic spin polarization modulates the spin density of MgPc, facilitating the LiPSs conversion kinetics in Li-S batteries.
- The MgPc@FCNT achieves ultra-low capacity decay rate under the high sulfur loading.

**Supplementary Information:**

The online version contains supplementary material available at 10.1007/s40820-023-01319-8.

## Graphical abstract

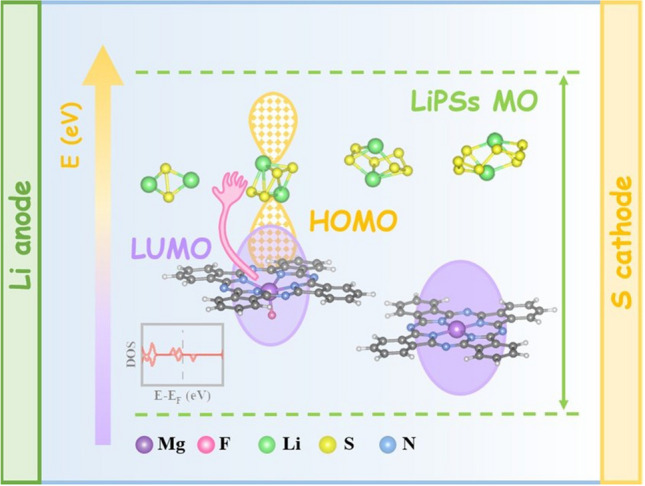

## Introduction

Since 1990s, the lithium-ion batteries (LIBs) have achieved commercialization in the energy storage market [1, 2]. However, the energy density of conditional LIBs is approximate to its limit of 300 Wh kg^−1^ [3]. The high-energy-density rechargeable batteries are urgently required to satisfy the ever-increasing demand [4]. Lithium-sulfur (Li–S) batteries are considered to be one of the most promising battery systems on account of the high specific density of 1675 mAh g^−1^ and energy density of 2600 Wh kg^−1^ [5, 6]. However, the scale manufacturing of Li–S batteries is severely hampered by their poor cycling stability and inferior rate capability deriving from the “shuttle effect” induced by the easily soluble lithium polysulfides (LiPSs) in the multi-step sulfur redox reactions [7, 8]. In order to solve these problems, the early strategies utilized the polar compounds [9–11] or metals [12] and electrolyte additives [13–15] to adsorb and catalyze the LiPSs intermediates. However, its high mass ratio restricts the improvement of the sulfur load and energy density [16]. Therefore, single-atom catalysts (SACs) have attracted extensive attention due to their maximized atomic utilization, flexible coordination configuration regulation and efficient catalytic activity [17–20]. In the past, metal-nitrogen doped carbon (M‒N‒C) catalysts were generally synthesized by high temperature pyrolysis, which was difficult to accurately control and identify its accurate structure. However, molecular complexes with nitrogen coordinated metal centers, for example metal phthalocyanine (MPc), have attracted the attention of researchers because of their clear structure and structural tunability [21, 22].

According to the reports, the *d*-block transition-metal SACs are heavily investigated to accelerate the redox kinetics in Li–S batteries when their catalytic activity can be effectively modulated based on the *d*-band center theory proposed by Nørskov [23–25]. Compared with the *d*-block SACs, the main group elements exhibit better stability because of the poor electronic flexibility [26]. The interaction between the adsorbate and the main group SACs will lead to a wide adsorption energy dispersion due to the broad *s/p*-band, resulting in the low catalytic reactivity of the main group SACs [27, 28]. Interestingly, the Mg‒N_4_ structure of chlorophyll molecule is closely related with the charge transfer state in the photosynthesis [29–31]. In addition, the main group Mg atoms in the cofactor enzymes play an important role in a variety of metabolic pathways and nucleic acid-related biochemical processes [32]. Inspired by the catalytic process, Chen et al. modulate the coordination number of Mg to adjust the occupied state of atom orbital and shift the energy levels alignment of the highest occupied molecular orbital (HOMO), optimizing its activity in oxygen reduction reaction (ORR) [33].

Concerning the macrocyclic compounds with a chelated metal ion, it is well known in the case of transition MPc that the central metal can adapt the conformations on the surface with the ion pointing toward and away from the substrate [34]. The axial displacement is accompanied by the spin state transition as well as the distinct changes in the molecular electronic structure [35]. In quantum mechanics, the transitions between two states that do not conserve spin are forbidden [36]. Considering the spin conservation for fast kinetics, the spin alignment in catalysts is critical for facilitating the spin-dependent reactions [37, 38]. The electronic spin polarization can promote the overlap-integral between the catalysts and the intermediates to enhance charge transfer, thus modifying the binding energy and potentially the reaction pathway [39]. Most active oxygen evolution reaction (OER) electrocatalysts usually exhibit anisotropic charge transport dependent on spin-dependent exchange interactions [40]. The dynamics of the charge transfer reaction can be partially controlled by the spin-dependent cooperative interaction. The spin-polarized electrons in catalysts promote the generation of spin holes by quantum spin exchange interactions (QSEI), which further promote the reaction kinetics [41]. Our previous research have confirmed that the axial bonding between central metal and ligand can effectively switch the spin state and upshift the energy levels of MPc and improve its catalytic activity [42]. In addition, electronegative axial halogen atoms can break the symmetry of M‒N_4_ structure, showing improved durability [43]. Therefore, the optimized electrocatalysts not only exhibit the highly active catalytic species but also accelerate the spin-selective electron transfer process.

In this work, we demonstrate that the LiPSs conversion kinetics can be significantly accelerated by manipulating the spin-polarized electrons of MgPc. The F-coordinated strategy is put forward to modulate the electron distribution and energy level alignment of Mg sites, which is implemented by anchoring MgPc on the fluorinated carbon nanotube matrix (denoted as MgPc@FCNT). According to the density functional theory (DFT) calculations, the electronic spin polarization in MgPc@FCNT not only increases the adsorption energy toward LiPSs intermediates but also facilitates the tunneling process of electron in Li–S batteries. As a result, the MgPc@FCNT in Li–S batteries shows long-term cycle stability with ultra-low-capacity attenuation of 0.029% per cycle at 2 C over 1000 cycles. Especially, even under a high sulfur loading of 4.5 mg cm^−2^, a high reversible area capacity of 5.1 mAh cm^−2^ can be maintained after 100 cycles.

## Experimental Section

### Synthesis of MgPc@FCNT and MgPc@CNT

30 mg of FCNT powder was dispersed in 30 mL of ethanol via sonication. 3 mg of MgPc dissolved in 10 mL of ethanol was then added to the suspension of FCNT. The mixture was under ultrasonication for 30 min and further stirred for 20 h at room temperature. The precipitate was collected and washed with ethanol and deionized water and then dried through a vacuum oven at 60 °C. MgPc@CNT sample was synthesized in the same way except for replacing FCNT with CNT.

### Preparation of Modified Separators

The modified separators were fabricated by the blade coating method. Take the preparation of MgPc@FCNT modified separator as an example, the slurry of MgPc@FCNT and poly(vinylidene fluoride) (with mass ratio of 7:1) was coated on the Celgard PP separator, and then dried in a vacuum oven at 60 °C overnight. The area loading of CNT, FCNT, MgPc@CNT, MgPc@FCNT is about 0.1 mg cm^−2^.

### Electrochemical Measurements

The cathode slurry was prepared by sulfur (70 wt%), Super C65 (20 wt%) and PVDF (10 wt%) dissolved in NMP. The CR2032 coin cells were assembled in an Ar-filled glove box. The ratio between electrolyte and sulfur is 15 μL mg^−1^ with the sulfur loading of ~ 1.0 mg cm^−2^. The diameter of each electrode disk is 14 mm. The electrolyte was 1 mol L^−1^ LiTFSI in DOL and DME (1:1 v/v) with 2 wt% LiNO_3_ as additive. The diameter of separator is 19 mm. CV was performed using an electrochemical workstation (CHI 660e) with a voltage window of 1.7‒2.8 V. EIS were obtained with a frequency range from 0.01 Hz to 100 kHz at different temperatures. Galvanostatic charge/discharge tests were carried out using a Neware Battery Testing System (CT-4008 T-5 V 10 mA/20 mA) with a voltage window of 1.7‒2.8 V (vs. Li^+^/Li).

### Materials Characterization

The morphological feature was characterized by scanning electron microscopy (JSM-7500) and high-resolution transmission electron microscopy (JEM-ARM300F). X-ray diffraction (XRD) patterns were measured through Cu Kα radiation (λ=1.5406 Å) at 30 kV and 20 mA with a scan rate of 5° min^−1^. Raman spectra were recorded by LabRAM HR Evo at ambient temperature. Nitrogen adsorption and desorption plots were tested using the standard degassing station of the Mac instrument and the 4-station fully automatic specific surface area analyzer of the American Micromeritics APSP2460 model. X-ray photoelectron spectroscopy (XPS) was performed by Thermo ESCALAB 280 system with the Kα radiation of Al (photon energy=1486.6 eV) anode as mono X-ray source. Fourier transform infrared (FT-IR) spectra was characterized with Nicolet 6700. Ultraviolet and visible spectra were performed by Lamda 1050. Electron paramagnetic resonance (EPR) spectra were recorded with microwave frequency of 9.84 GHz, microwave power of 2.00 mW, and modulation amplitude of 4.00 Gauss at temperature of 298 K. UPS were recorded by PHI 5000 VersaProbe III (Scanning ESCA Microprobe) SCA (Spherical Analyzer). The soft X-ray absorption spectroscopy (sXAS) experiments were performed at beamline 02B02 of the SiP ME2 platform at the Shanghai Synchrotron Radiation Facility (SSRF). The bending magnet beamline provided photons with energy range from 50 to 2000 eV.

## Results and Discussion

### Structure Characterization of MgPc@FCNT

**Fig. 1:**
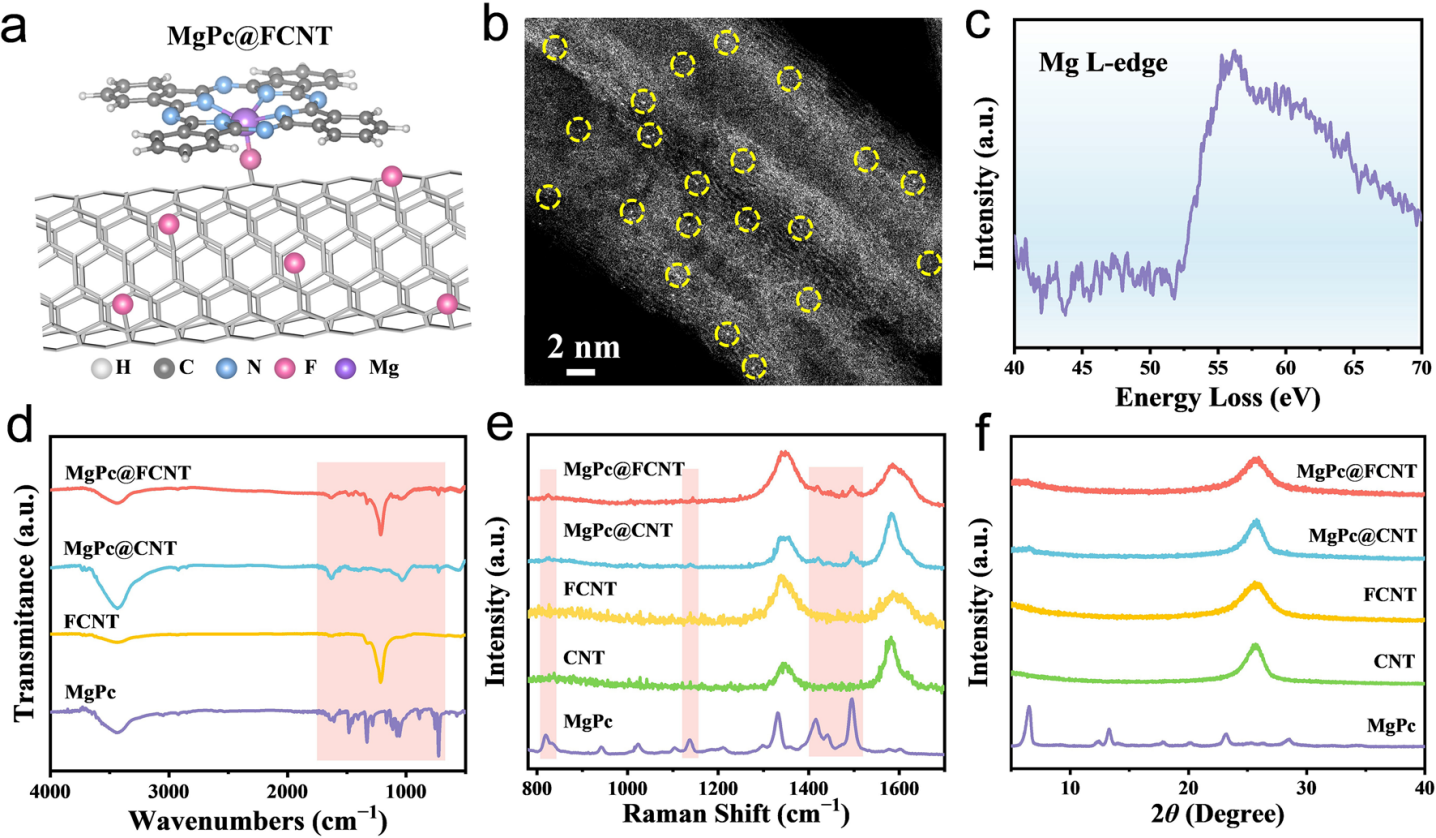
**a** Schematic illustration of the fabricated single-metal active sites. **b** HAADF-STEM image of MgPc@FCNT. **c** EELS spectrum of Mg L-edge. **d** FT-IR spectra **e** Raman spectra and **f** XRD patterns of MgPc@FCNT, MgPc@CNT, FCNT and MgPc

The main group electrocatalysts with uniformly dispersed active site were fabricated by loading MgPc on the surface of FCNT and CNT (Fig. 1a). The centrifugal supernatant of the mixed solution is clear and transparent for the MgPc@FCNT and MgPc@CNT (Fig. S1). Therefore, it is speculated that MgPc can be successfully loaded on FCNT and CNT. The prepared MgPc@FCNT and MgPc@CNT still maintain their morphology of nanotube after the loading of MgPc (Figs. S2 and S3). In addition, no obvious aggregation is observed on the surface of MgPc@FCNT through high-resolution transmission electron microscopy (HRTEM) (Fig. S4). The spherical aberration-corrected high angle annular dark field scanning transmission electron microscope (HAADF-STEM) image shows the isolated bright spots on the walls of FCNT, confirming the successful loading of MgPc (Figs. 1b and S5). The L-side signal of Mg (Fig. 1c) can be obtained from the electron energy loss spectroscopy (EELS), further verifying that Mg atoms are uniformly dispersed on FCNT. The scanning transmission electron microscopy energy-dispersive X-ray spectroscopy (STEM-EDS) analysis of element mapping further supports the uniform distribution of Mg and N along the FCNT, suggesting the presence of MgPc (Fig. S6). The inductive coupled plasma mass spectrometer (ICP-MS) test reflects that the content of Mg atom is around 0.36%, which is consistent with EDS and XPS results (Table S1).

The molecular structure of the prepared samples was characterized by FT-IR spectroscopy. Both of the MgPc@FCNT and MgPc@CNT showed typical CN-heterocyclic tensile vibration mode (shadded area), indicating that MgPc has been successfully loaded on the nanotubes (Figs. 1d, S7 and Table S2). The Raman spectra of MgPc@FCNT, MgPc@CNT, FCNT, and CNT show two broad peaks at ~1340 and ~1585 cm^−1^, belonging to the D and G bands, respectively. The intensity of *I*_D_/*I*_G_ for MgPc@FCNT and FCNT is higher than that of MgPc@CNT and CNT (Fig. 1e), which may be ascribed to the relatively abundant defects for FCNT [44]. In addition, the stretching modes of the C‒N_m_‒C and C_β_‒C_β_ at 1415 and 1495 cm^−1^ were observed for MgPc@FCNT and MgPc@CNT, which further proved the combination between MgPc and the nanotube substrates. The N_2_ adsorption and desorption isotherms were measured, and the pore size distribution for MgPc@FCNT and MgPc@CNT was around 30 nm (Fig. S8), which exclude the influence of the microstructure on the performance difference. According to the XRD patterns, there exist no characteristic peaks of MgPc in the MgPc@FCNT and MgPc@CNT samples (Fig. 1f), indicating that there is no agglomeration of MgPc on the walls of the nanotube [45].

### Coordination Environment and Electronic Structure Regulation

**Fig. 2:**
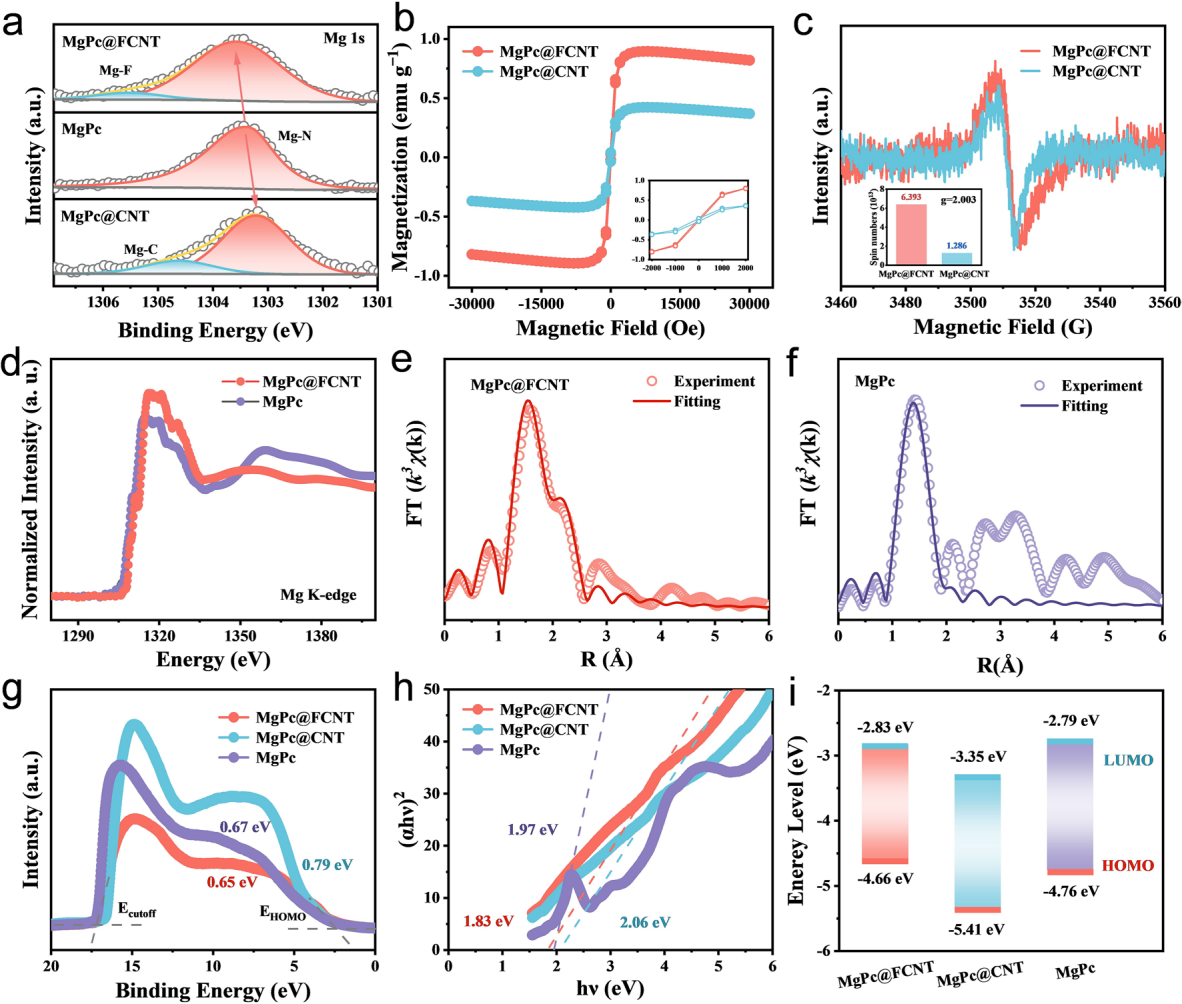
**a** XPS spectra of Mg 1*s* for MgPc@FCNT, MgPc@CNT and MgPc. **b** Magnetization curves (M-H) of MgPc@FCNT and MgPc@CNT. The inset shows hysteresis loop with enlarged x-axis. **c** EPR spectra of MgPc@FCNT, MgPc@CNT. The inset shows the spin numbers. **d** Mg K-edge XANES of MgPc@FCNT and MgPc. FT-EXAFS spectra and fitting curves of **e** MgPc@FCNT and **f** MgPc. **g** UPS spectra **h** Tauc plots and **i** HOMO/LUMO level alignment obtained through experiments

XPS was used to further study the valence and coordination environment of Mg sites from the MgPc@FCNT and MgPc@CNT. Based on the XPS spectra, MgPc@FCNT is mainly composed of C, N, F, and Mg elements (Fig. S9). In order to explore the atomic configuration, the Mg 1*s*, N 1*s*, and F 1*s* spectra were deconvoluted into a series of peaks. The Mg 1*s* spectra of MgPc@FCNT (Fig. 2a) could be deconvoluted into Mg‒N (1303.4 eV) and Mg‒F (1305.5 eV) when that of MgPc@CNT corresponds to Mg‒N and Mg‒C (1304.5 eV). Compared with MgPc, the Mg 1*s* spectrum shift toward the higher energy for MgPc@FCNT and toward lower energy for MgPc@CNT. The high-resolution F 1*s* spectrum can be deconvoluted into C‒F (688.6 eV) and Mg‒F (684.8 eV), which further verifies the Mg‒F for MgPc@FCNT (Fig. S10). The signal evolution of N 1*s* peak is also prominent. It is generally considered that the strong molecule-substrate interaction would cause a peak splitting, while the weak interaction would cause only a peak shift. Actually, a new peak above 400 eV was observed for all the samples. MgPc@FCNT shows the most intense new peak at ~400.5 eV, indicating a strong interaction between MgPc and FCNT [34]. The magnetic properties were measured by superconducting quantum interference device (SQUID), showing typical hysteresis loops and saturated magnetic fields (Fig. 2b). Both MgPc@FCNT and MgPc@CNT exhibit ferromagnetism at room temperature [46]. The saturated magnetization and coercivity for MgPc@FCNT are significantly greater than those of MgPc@CNT. To explore the source of magnetism of both nanotubes, EPR spectra were further measured. The EPR spectra of MgPc@FCNT and MgPc@CNT display the g value of 2.003 which was consistent with that of MgPc (Fig. S11). However, compared with MgPc@CNT, the signal of MgPc@FCNT is significantly intensified and broadened (Fig. 2c), suggesting the increased concentration of magnetic substance [47, 48]. By quantifying their free spin electron numbers, MgPc@FCNT shows a significantly higher spin concentration, indicating more unpaired electrons [49]. These changes in molecular magnetism are correlated with their changes in the electronic structure.

Furthermore, the local environment of Mg sites was investigated by X-ray absorption fine structure (XAFS) spectra. Figure 2d presents the X-ray absorption near-edge structure (XANES) spectra. The K-edge energy level of Mg for MgPc@FCNT shifts to the positive direction compared to MgPc. This data suggests that the valence state of Mg for MgPc@FCNT increases. The k^3^-weighted Fourier-transformed extended X-ray absorption fine structures (FT-EXAFS) and quantitative least-squares curve-fitting verify the coordination structure of MgN_4_F (Fig. 2e, f and Table S3). This axial Mg‒F bonding changes the ligand fields and promotes the electron exchange between F and Mg atoms.

In order to determine the electronic structure, the energy level alignment of molecular orbital for MgPc@FCNT, MgPc@CNT and MgPc were determined by UV spectroscopy. The work function (*φ*) can be calculated from the cut-off edge of the secondary electron among the high energy region through the ultraviolet photoelectron spectroscopy (UPS) diagram. The *φ* decreases from 4.62 eV for MgPc@CNT to 4.01 eV for MgPc@FCNT (Fig. S12), clearly manifesting that the axial electron-withdrawing group F could raise the Fermi level (*E*_F_) and promote the electron transport [39, 50]. The first peak among the low energy region is related to the energy level of HOMO. Compared with MgPc, the HOMO of MgPc@FCNT is slightly downward shifted by 0.02 eV, while that of MgPc@CNT is upward shifted by 0.12 eV (Figs. 2g and S13). The results indicate the presence of a lower tunneling barrier for MgPc@FCNT [51, 52]. By the UV–vis spectra, the absorption edges of MgPc@FCNT and MgPc@CNT are similar (Fig. S14), which may be ascribed to their similar spatial configurations. According to the band gap diagram converted by Kubelka Munk equation, MgPc@FCNT shows the smallest band gap of 1.83 eV (Fig. 2h) among the three samples. According to the frontier molecular orbital (FMO) theory, the narrow band gap delivers improved conductivity, facilitating the electrochemical processes [53]. The energy level of LUMO can be speculated from the values of HOMO and band gap. The results show that the MgPc@FCNT demonstrates a decreased energy level alignment of LUMO compared with that of MgPc (Fig. 2i), which is consistent with the calculation results (Fig. S15).

### Evaluation of Catalytic Activity

**Fig. 3:**
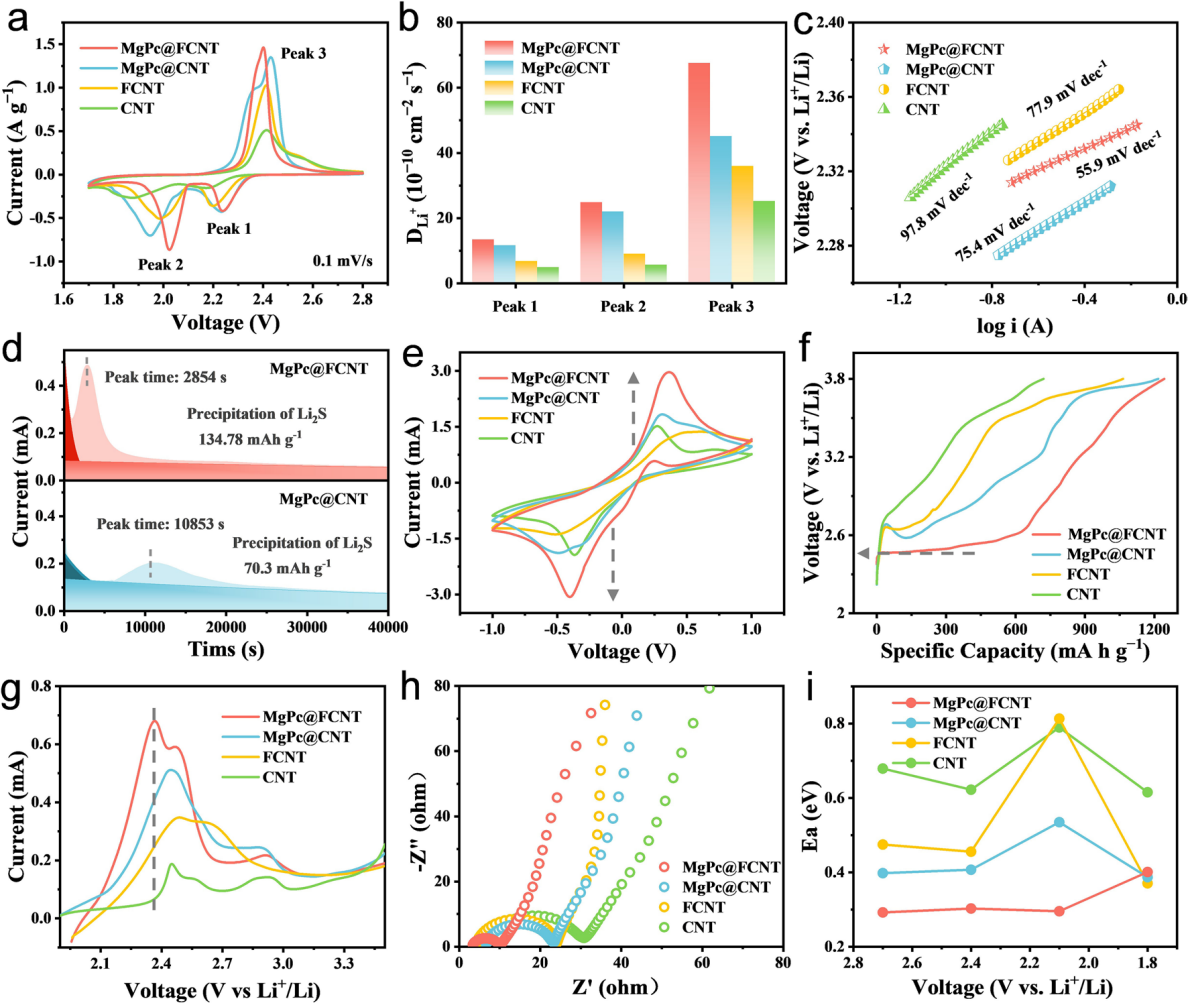
**a** CV curves. **b** Li ion diffusion coefficient derived from CV profiles. **c** Tafel slope of peak 3. **d** Potentiostatic discharge profiles at 2.05 V. **e** CV curves of symmetric cells. **f** Galvanostatic charge curves and **g** LSV curves. **h** EIS plots of MgPc@FCNT, MgPc@CNT, FCNT and CNT. **i** Activation energy barriers at a given discharge voltage for MgPc@FCNT, MgPc@CNT, FCNT and CNT

Electrochemical kinetics experiments were carried out to study the electrocatalytic effect of a series of samples in sulfur reduction reaction and sulfur evolution reaction. Cyclic voltammetry (CV) measurements were performed at scanning rates of 0.1‒0.5 mV s^−1^ (Fig. S16) to investigate the redox characteristics. During the discharge process, the two characteristic cathodic peaks could be attributed to the formation of soluble LiPSs (2.2‒2.3 V) and insoluble Li_2_S_2_/Li_2_S (1.9‒2.1 V), respectively [54]. Meanwhile, the anodic peak at 2.4 V corresponds to the decomposition of Li_2_S during the charge process (Fig. 3a). It can be seen that MgPc@FCNT shows the greater current response and lower polarization voltage than the other three catalysts due to the superior electrocatalytic activity of the unique Mg‒N_4_F sites [55]. The current densities for the cathodic and anodic peaks are linear with the square root of scanning rate, indicating the diffusion limiting process (Fig. S17). Therefore, the classical Randles Sevcik equation can be used to evaluate the diffusion characteristics of Li ions: *I*_p_ = (2.69 × 10^5^) n^1.5^SD^0.5^Cν^0.5^, where *I*_p_ is the current density of peak, *n* is the number of charge transfer, *S* is the electrode area, *D* is the diffusion coefficient of Li ions, *C* is the concentration of Li ions in the electrode, and *ν* is the scan rate [56]. MgPc@FCNT shows the highest Li ion diffusion coefficient for the three redox peaks (Fig. 3b), which confirms that the axial coordination effect of electron-withdrawing group F promotes the LiPSs conversion in Li–S batteries. In addition, the Tafel slope corresponding to each peak can be calculated based on the linear sweep voltage (LSV) curve, which is an index to evaluate the catalytic activity [57]. The smaller Tafel slope exhibits, the better catalytic ability achieves. The Tafel slope for MgPc@FCNT is smaller than other samples (Figs. 3c and S18), indicating the best catalytic activity. During the process of Li_2_S deposition, Li_2_S_6_ solution was used as electrolyte. After discharging to 2.06 V via galvanostatic method, potentiostatic method is operated at 2.05 V (Figs. 3d and S19). The typical potentiostatic I-t curves may be separated into three regions by two exponential functions: the reduction of Li_2_S_8_ and Li_2_S_6_ (dark areas), and the precipitation of Li_2_S (light area) [58]. According to the integral area, the Li_2_S deposition capacity for MgPc@FCNT (134.78 mAh g^−1^) is significantly higher than that of MgPc@CNT (70.3 mAh g^−1^). The higher Li_2_S capacity suggests the improved kinetics in Li–S batteries. Therefore, this result confirms that MgPc@FCNT is the most advantageous in terms of reaction kinetics and catalytic activity for the reduction of LiPSs to Li_2_S. The CV curves of the symmetrical cells were collected in Li_2_S_6_ electrolyte with a scanning rate of 1 mV s^−1^. All cells show a pair of symmetrical redox peaks (Fig. 3e). Compared with the MgPc@CNT, FCNT and CNT, MgPc@FCNT exhibits the strongest oxidation–reduction peaks, indicating the optimized electrochemical kinetics in Li–S batteries.

The catalytic efficiency for a series of catalysts during the charging process was further surveyed by the galvanostatic charge method. The potential response of MgPc@CNT, FCNT and CNT sharply increase to ~2.7 V and gradually slow down, meaning that Li_2_S began to dissociate at ~2.7 V for the three samples. However, MgPc@FCNT delivers a low dissociated potential of 2.46 V (Fig. 3f). In addition, MgPc@FCNT contributes to a specific capacity of 630 mAh g^−1^, which is higher than other samples, suggesting that MgPc@FCNT facilitates the dissociation process of Li_2_S. This result proved that the fabricated Mg‒N_4_F sites could effectively reduce the reaction energy barrier in sulfur evolution reaction. In LSV test, the peak current of MgPc@FCNT, locating at the lower potential than the others, is higher than the others, which prove the optimized catalytic activity for Li_2_S dissociation (Fig. 3g). In addition, by the EIS curves (Fig. 3h), MgPc@FCNT shows the smallest charge transfer resistance, indicating the improved electrochemical kinetics. In order to quantify the dynamics characteristics in SRR, the activation energy barriers at a series of voltage were determined (Figs. S20‒S23). By fitting the charge transfer resistance under different temperatures by Arrhenius equation (Fig. S24), the logarithm of the reciprocal of the charge transfer resistance is linear with the reciprocal of the absolute temperature, from which *E*_a_ can be obtained at each measured voltage. Under the voltage of 2.4‒2.1 V, S_8_ molecule reacts with Li^+^ to form long-chain Li_2_S_8_, which is then transformed into a series of short-chain LiPSs through the fracture of S‒S bond. Li_2_S_6_ and Li_2_S_4_ are converted into insoluble Li_2_S_2_/Li_2_S products at 2.1‒1.8 V. The initial cleavage of ring S_8_ molecule is considered to be a relatively facile process, while the conversion of LiPSs to insoluble products is particularly slow, which is considered to be the slowest kinetic reaction process [17]. At 2.1 V, *E*_a_ for MgPc@FCNT, MgPc@CNT, FCNT and CNT was 0.29, 0.69, 0.81 and 0.78 eV, respectively (Fig. 3i), of which MgPc@FCNT shows the lowest *E*_a_. This result further indicates that MgPc@FCNT contributes to the fastest reaction kinetics.

### Evaluation of Electrochemical Performance

**Fig. 4:**
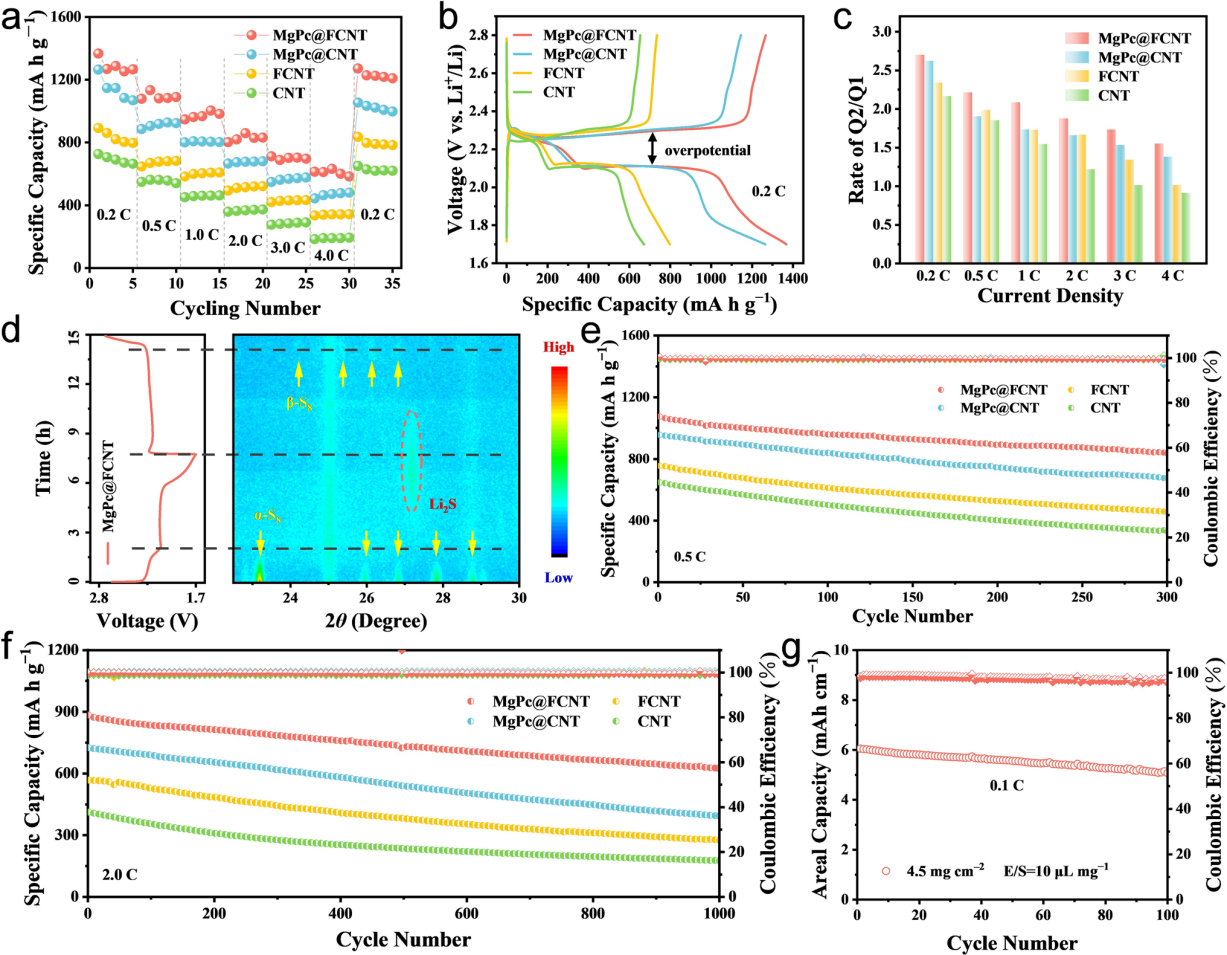
**a** Rate performance for MgPc@FCNT, MgPc@CNT, FCNT and CNT. **b** GCD profiles at 0.2 C. **c** Q2/Q1 at different rates. **d** 2D pseudo-color in-situ XRD patterns of MgPc@FCNT during the GCD processes. Cycling performance **e** at 0.5 C and **f** at 2 C. **g** Cycling performance with high sulfur loading at 0.1 C

The rate capacity and cycle behavior were researched by assembling the CR2032 coin cells with separators modified by different catalysts. The coated MgPc@FCNT layer adheres firmly on the polypropylene (PP) membrane and maintains high stability without mechanical delamination under bending, wrinkling or immersion in electrolyte (Fig. S25). When the mass loading of sulfur was about 1 mg cm^−2^, the MgPc@FCNT provides specific capacities of 1367, 1133, 1003, 859, 711, 631 mAh g^−1^ at 0.2, 0.5, 1, 2, 3 and 4 C, respectively (Figs. 4a and S26). Compared with the other catalysts, MgPc@FCNT performs the highest capacity retention rate of 62.8% at 2 C compared with 0.2 C (Fig. S27), which is attributed to its effective inhibition of LiPSs and the acceleration of electrocatalytic conversion kinetics. In the galvanostatic charge–discharge (GCD) curve of 0.2 C, the high discharge platform and low discharge platform correspond to the conversion reaction of LiPSs and the formation of insoluble Li_2_S_2_/Li_2_S, respectively (Fig. 4b). The ratio of the capacity contributing to the first and second voltage platform is marked as Q2/Q1, which represents the catalytic performance on the LiPSs conversion. Among the samples, MgPc@FCNT shows the highest Q2/Q1 value from 0.2 to 4 C (Fig. 4c), which confirms the best conversion kinetics in sulfur redox reaction.

To further investigate the sulfur evolution mechanism and redox kinetics of sulfur species during the GCD reaction, in-situ XRD analysis was performed (Figs. 4d and S28). Diffraction peaks of *α*-S_8_ (23.184°, 25.912°, 26.833°, 27.826°, and 28.782°) were detected at the open circuit potential. As the discharge progress, the sharp peaks of *α*-S_8_ gradually disappeared. Compared with MgPc@CNT, the peaks of *α*-S_8_ for MgPc@FCNT rapidly disappear, indicating the rapid conversion kinetics from *α*-S_8_ to LiPSs. When discharged to 2.05 V, a diffraction peak of Li_2_S (27.171°) appeared, proving the rapid reduction rate of LiPSs and nucleation of Li_2_S. During the charging process, the peak intensity of Li_2_S decreased gradually, proving that MgPc@FCNT can continuously promote the nucleation and conversion of Li_2_S, which is consistent with the experimental results of Li_2_S deposition. At the end of charging, the formation of *β*-S_8_ meant that MgPc@FCNT improved the reversibility of sulfur cathode during the GCD processes. The cycle performance of Li–S batteries was evaluated at 0.5 C (Fig. 4e). MgPc@FCNT provides 837.5 mAh g^−1^ after 300 cycles, which is much higher than that of MgPc@CNT (676.5 mAh g^−1^), FCNT (458.5 mAh g^−1^) and CNT (330.6 mAh g^−1^). In addition, the long-term stability at 2 C was studied over 1000 cycles (Fig. 4f). The ultra-low capacity decay rate of 0.029% can be achieved for MgPc@FCNT (Fig. S29). The morphology of the cathode have been investigated after 100 cycles test (Fig. S30). The MgPc@FCNT shows a uniform and dense deposition layer, while that of MgPc@CNT shows a coarsened deposition layer. This result indicated that the MgPc@FCNT effectively catalyzed the conversion reaction of LiPSs [59, 60]. Furthermore, the cycling performance under high sulfur loading of 4.5 mg cm^−2^ was studied (Fig. 4g). MgPc@FCNT can provide high area capacity of 5.1 mAh cm^−2^ after 100 cycles indicating the potential commercial application compared with other materials [61, 62].

### Analysis of Catalytic Mechanism

**Fig. 5:**
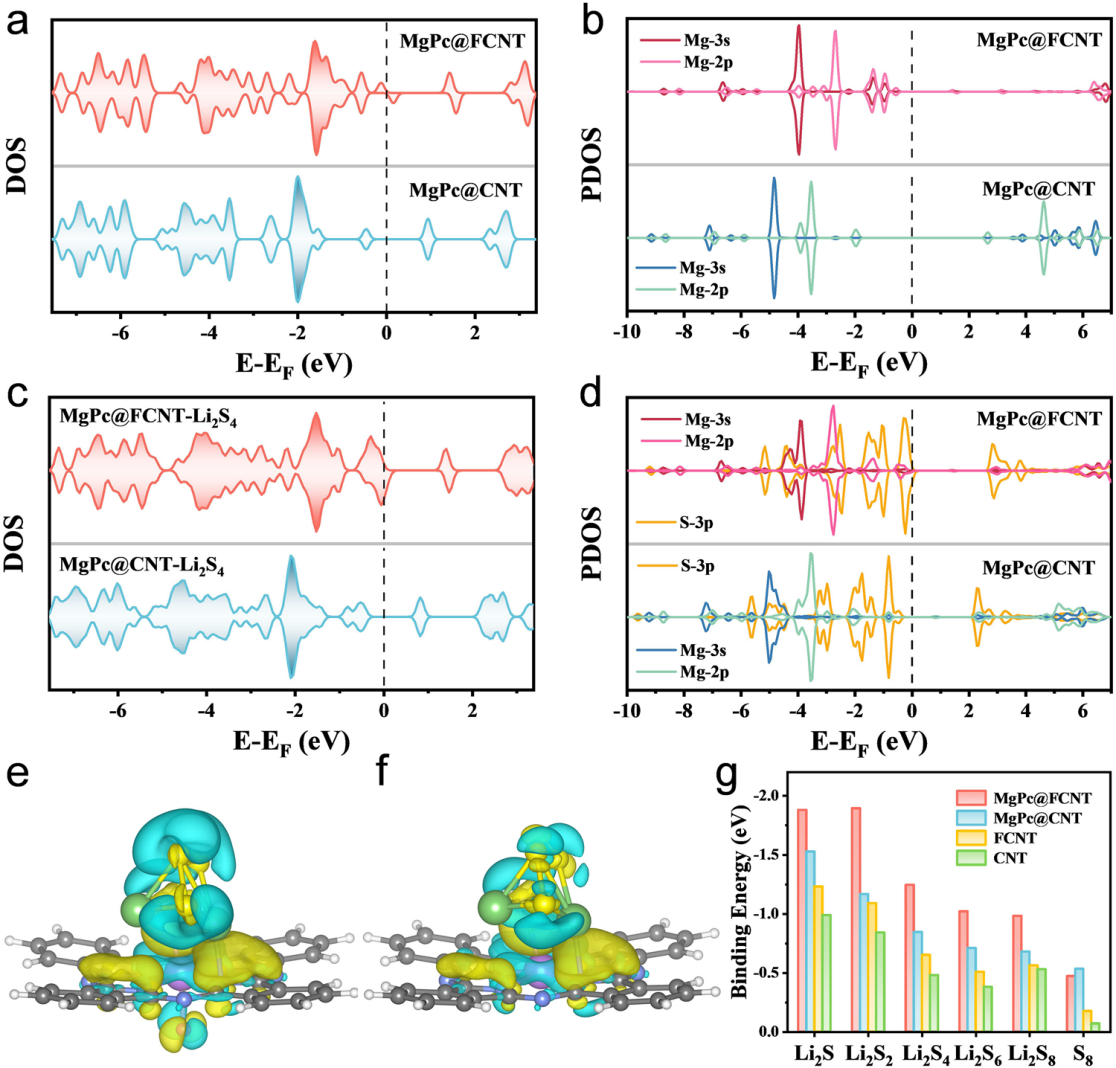
The calculated DOS for MgPc@FCNT and MgPc@CNT **a** before and **c** after the adsorption of Li_2_S_4_. The PDOS of 3*s*/2*p* orbitals of Mg **b** before and **d** after the adsorption of Li_2_S_4_. Charge distribution diagrams of **e** MgPc@FCNT and **f** MgPc@CNT. **g** Binding energies between MgPc@FCNT, MgPc@CNT, FCNT, CNT and Li_2_S_n_ (n = 1, 2, 4, 6, 8)/S_8_

The axial displacement of Mg sites leads to the electronic spin polarization, affecting the chemisorption, band gap and charge transport properties [63]. DFT calculations were performed to gain an atomic-level understanding on the spin polarization and the interaction between LiPSs and Mg sites (Fig. S31). The structural models of MgPc@FCNT and MgPc@CNT were optimized, respectively (Fig. S32). The bond angle of N‒Mg‒N from MgPc@FCNT was calculated to be 142° (179° for MgPc), indicating the geometrical structure distortion of the Mg center in MgPc@FCNT. The density of states (DOS) illustrate that the Fermi level of MgPc@FCNT passes through the valence band suggesting its ferromagnetic metallic properties [64]. A wide band gap of MgPc@CNT indicates its semiconductor characteristics with poor electrical conductivity (Fig. 5a). Projected density of states (PDOS) analysis of Mg-3*s*/2*p* orbitals reveals that charge transfer between F and Mg leads to increased magnetic moments for Mg, thereby increasing the spin polarization of MgPc@FCNT (Fig. 5b). When Li_2_S_4_ was adsorbed on Mg site of MgPc@FCNT (Fig. 5c), the occupation state near the Fermi level increases indicating the strong covalent bonding. According to the molecular orbital theory, the stronger bonding leads to the firmer adsorption between MgPc@FCNT and Li_2_S_4_. As well, the calculation indicates that compared to MgPc@CNT with anti-parallel couplings, the MgPc@FCNT with spin alignment has a higher spin density on the Li_2_S_4_. The magnetic moment of MgPc@CNT is 0 μB and of MgPc@FCNT is 0.885 μB with spin alignment, which indicates a FM ligand hole in MgPc@FCNT due to charge transfer between axial coordination F and Mg. A concomitant increment of the orbitals hybridization associated with FM ligand holes will facilitate spin-selected charge transport [65], leading to the decreased electron–electron repulsion for the spin exchange between MgPc@FCNT and the adsorbed Li_2_S_4_. As shown in the PDOS (Fig. 5d), the overlap between Mg-3*s*/2*p* orbitals and S-3*p* orbitals increases for MgPc@FCNT, leading to the stronger *s-p* and *p-p* hybrid orbitals [39]. In addition, the adsorption energies for Li atom on MgPc@FCNT and MgPc@CNT are −2.57 and −1.34 eV, while those for S atom is are −3.84 and −1.69 eV, respectively (Fig. S33). This result suggests that S is inclined to adsorb on Mg site. Charge distribution diagrams for Li_2_S_4_ adsorbed on MgPc@FCNT and MgPc@CNT show umbrella-shaped charge density distribution (Fig. 5e, f). It is observed that MgPc@FCNT shows charge accumulation on the S atom and electron density depletion on the Mg atom. In addition, more charge depletion occurred at the far end of S, indicating an enhanced charge transfer ability. In addition, when Li_2_S and Li_2_S_8_ adsorbed on MgPc@FCNT, there will be shorter Mg–S bond compared with those of MgPc@CNT (Table S4), indicating the stronger adsorption capacity. The adsorption ability toward all sulfur species on MgPc@CNT, FCNT and CNT is significantly lower than that of MgPc@FCNT, indicating the outstanding adsorption ability for MgPc@FCNT (Figs. 5g and S34, S35). Therefore, the spin-polarized electron exchange between the ferromagnetic MgPc@FCNT and adsorbed sulfur species is ferromagnetic-exchange-like under the principle of spin angular momentum conservation. This quantum spin-exchange interactions optimize the reaction kinetics in Li–S batteries.

## Conclusions

In summary, we employed FCNT to anchor MgPc and achieved the accurate fabrication of Mg SACs with axial displacement. Compared with MgPc, the axial coordination regulation activates the main group Mg SACs due to the optimized spin density, showing highly catalytic activity in Li–S batteries. DFT calculations demonstrated that spin-polarized electron in MgPc@FCNT not only increases the adsorption energy toward LiPSs intermediates but also facilitates the tunneling process of electron in Li–S batteries. As a result, the MgPc@FCNT provides an initial capacity of 6.1 mAh cm^−2^ even when the high sulfur loading is 4.5 mg cm^−2^, and still maintains 5.1 mAh cm^−2^ after 100 cycles. This strategy demonstrates that the rational spin engineering can optimize the catalytic activity and expands the potential applications of the main group metal SACs in Li–S batteries.

## Supplementary Information

Below is the link to the electronic supplementary material.

Supplementary file1 (PDF 2591 kb)
