# Supplementary material for: Quantum Spin Exchange Interactions to Accelerate the Redox Kinetics in Li–S Batteries
Source: Nanomicro Lett. 2024 Jan 29;16:100. doi: 10.1007/s40820-023-01319-8 (PMC10825106; doi:10.1007/s40820-023-01319-8)
Supplement: Supplementary file 1 — Supplementary file1 (PDF 2591 kb) [file 40820_2023_1319_MOESM1_ESM.pdf]

Supporting Information for

Quantum Spin Exchange Interactions to Accelerate the Redox Kinetics in Li-S Batteries

Yu Du[1], Weijie Chen[1], Yu Wang[1], Yue Yu[1], Kai Guo[1], Gan Qu[1,*] and Jianan Zhang[1,*]

[1]Key Laboratory of Advanced Energy Catalytic and Functional Materials Preparation of Zhengzhou City, College of Materials Science and Engineering, Zhengzhou University, Zhengzhou 450001, P. R. China

*Corresponding authors. E-mail: gqu@zzu.edu.cn (G. Qu), zjn@zzu.edu.cn (J. Zhang)

S1 Computational Methods

The DFT calculations were performed on Vienna ab initio Simulation package (VASP) [S1, S2] with projector augmented wave (PAW) pseudopotential [S3, S4]. The exchange-correlation interaction was described by Perdew-Burke-Ernzerhof (PBE) functional within the spin-dependent generalized gradient approximation (GGA) [S5]. The kinetic energy cut-off was set to 500 eV, and the dispersion corrections in Grimme's scheme (DFT-D3) was utilized to treat the long-range van der Waals interactions [S6]. For structure optimization, the Brillouin zone was sampled by Gamma-centered k-point with 3 × 3 × 1, and the convergence criterion of force and energy were set to 0.01 eV Å^{-1} and 10^{-5} eV, respectively [S7]. For electronic structure calculation, the total energy was converged to 10^{-7} eV.

S2 Supplementary Figures and Tables

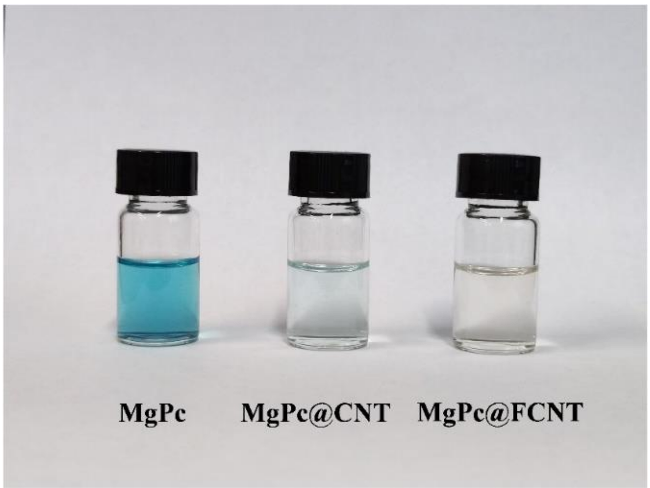

Fig. S1 The supernatant of MgPc, MgPc@CNT and MgPc@FCNT after the centrifugation

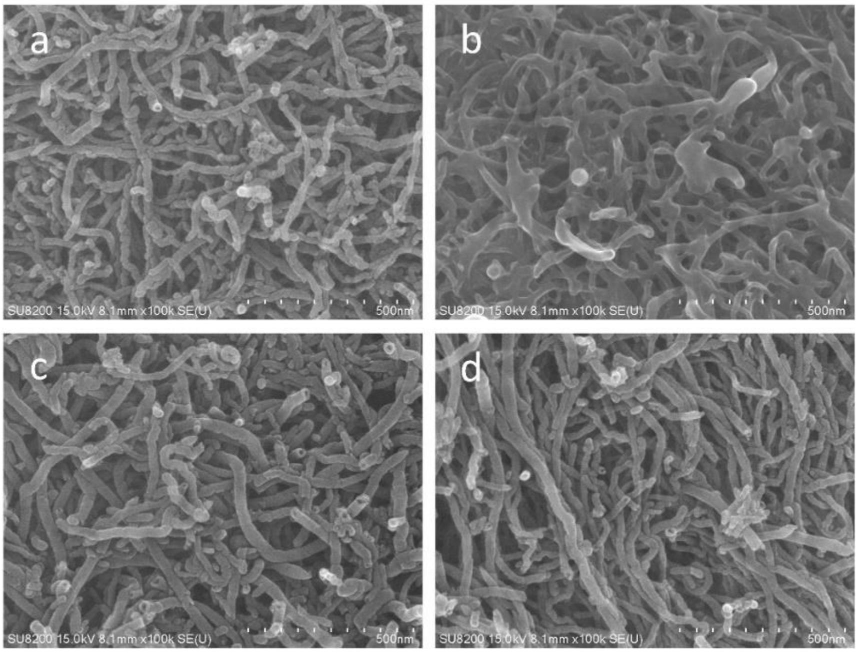

Fig. S2 SEM images of **a** CNT, **b** FCNT, **c** MgPc@CNT and **d** MgPc@FCNT

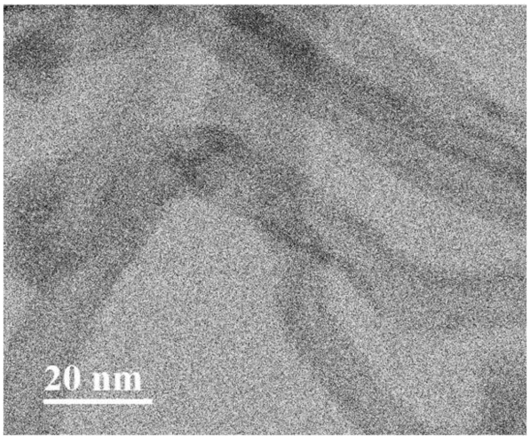

Fig. S3 TEM images of MgPc@FCNT

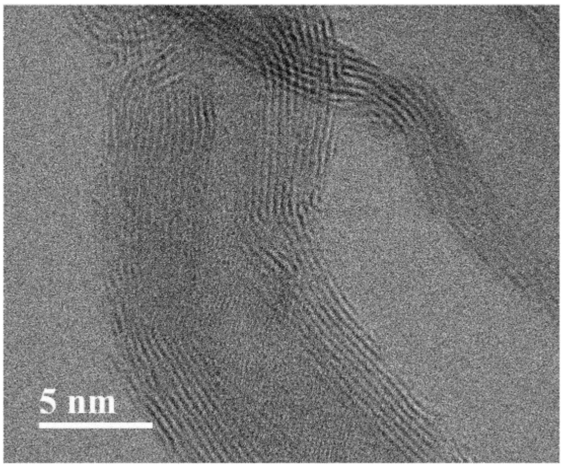

Fig. S4 HRTEM images of MgPc@FCNT

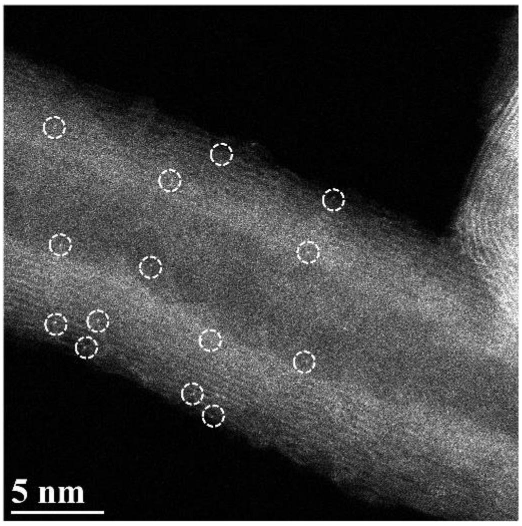

Fig. S5 Cs-HAADF-STEM images of MgPc@FCNT

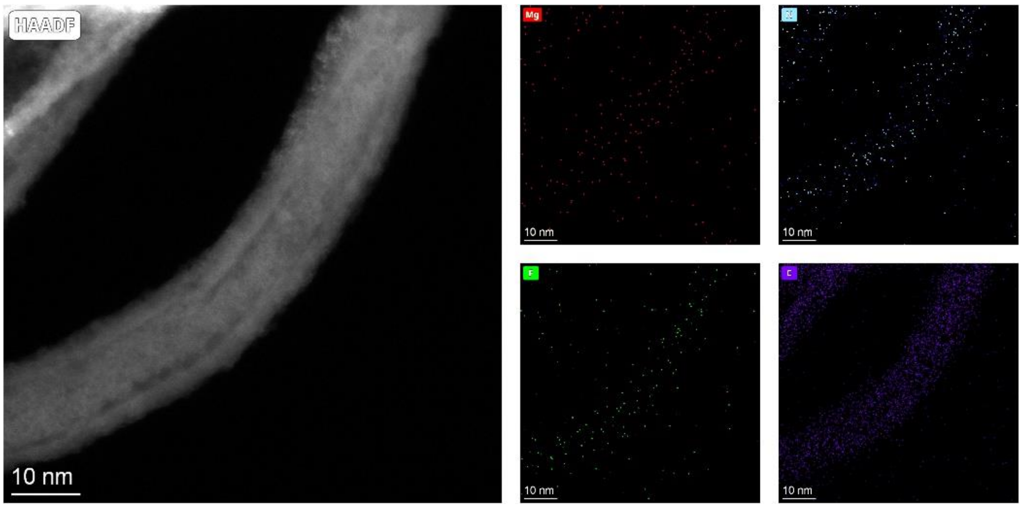

Fig. S6 STEM-EDS elemental mapping of MgPc@FCNT

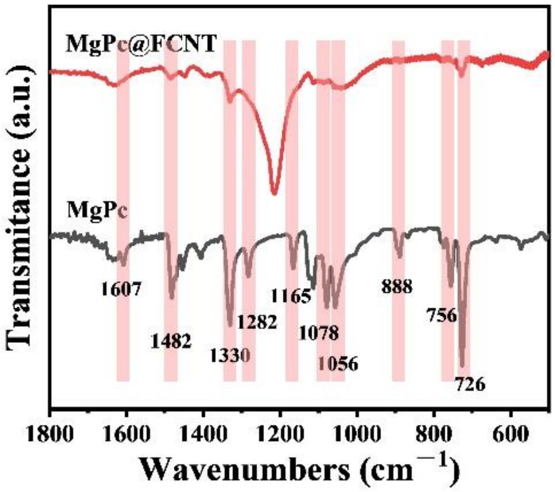

Fig. S7 FT-IR spectra of MgPc@FCNT and MgPc

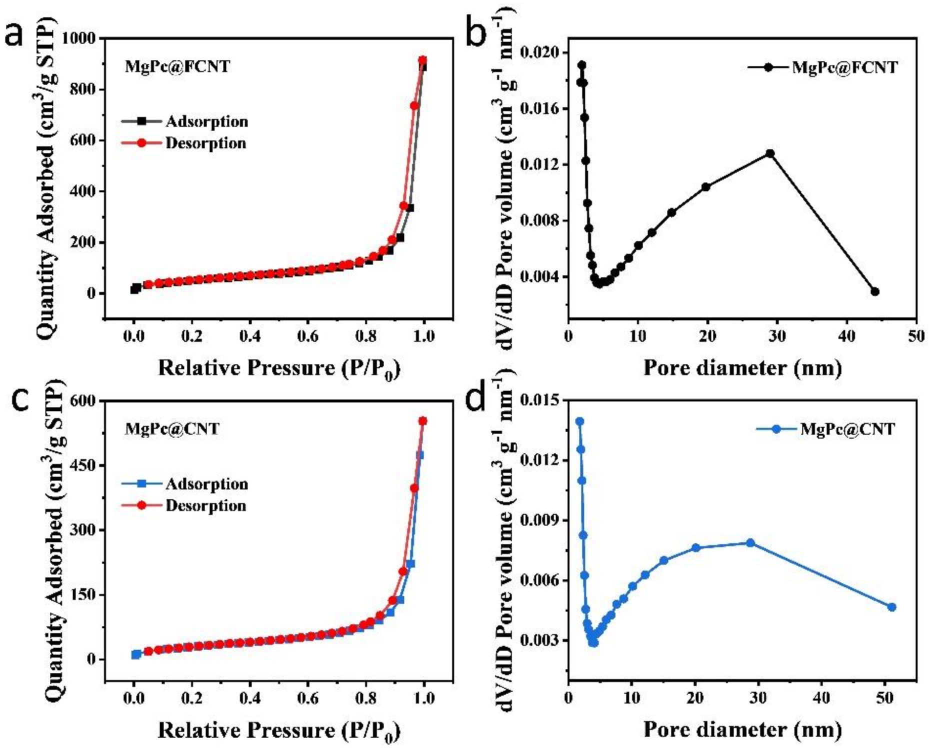

Fig. S8 Nitrogen adsorption and desorption isotherm and pore size distribution curves of **a, b** MgPc@FCNT and **c, d** MgPc@CNT

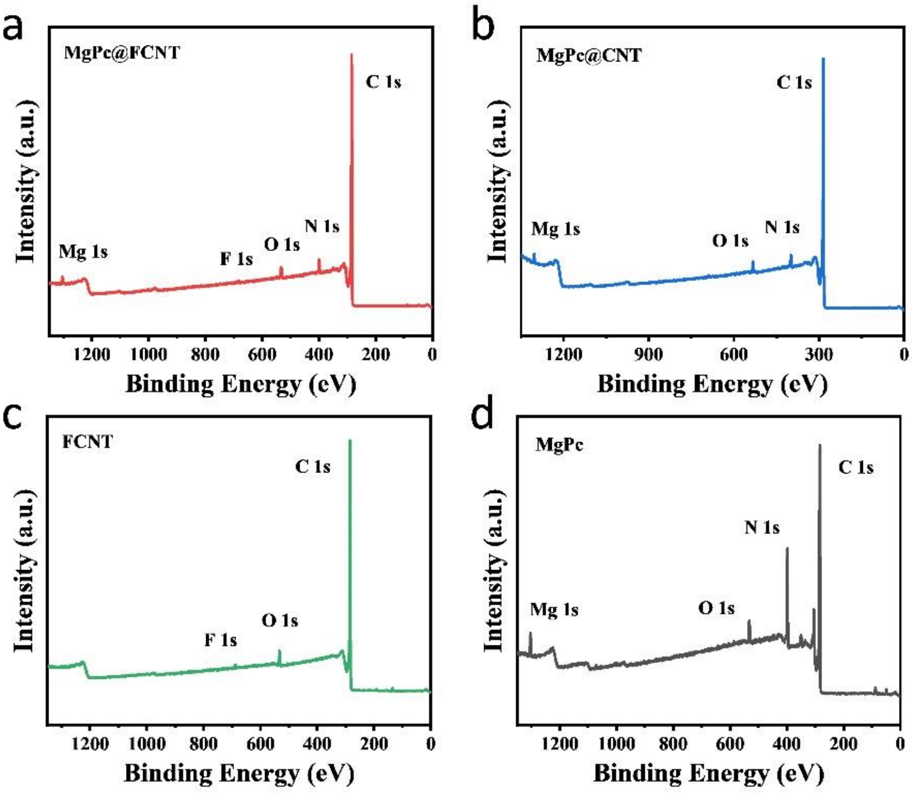

Fig. S9 XPS survey of **a** MgPc@FCNT, **b** MgPc@CNT, **c** FCNT and **d** MgPc

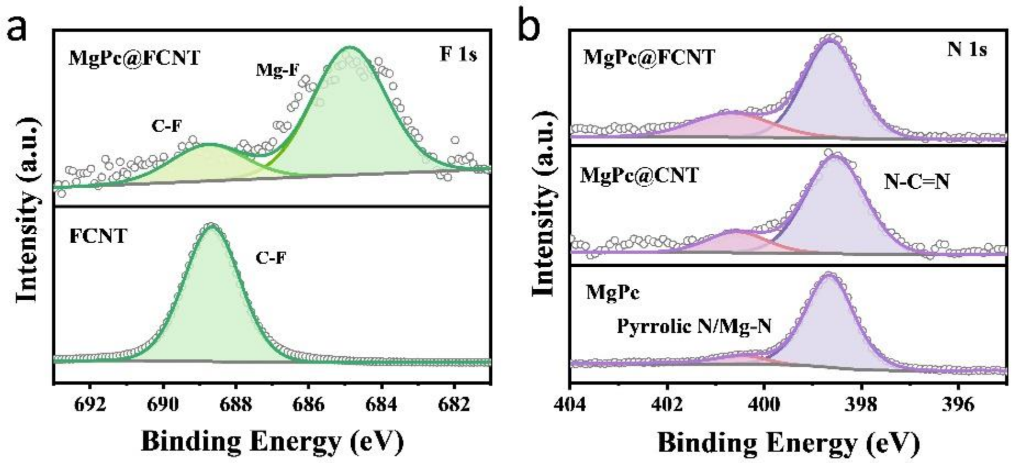

Fig. S10 XPS spectra of MgPc@FCNT, MgPc@CNT, FCNT and MgPc. **a** F 1s spectrum, **b** N 1s spectrum

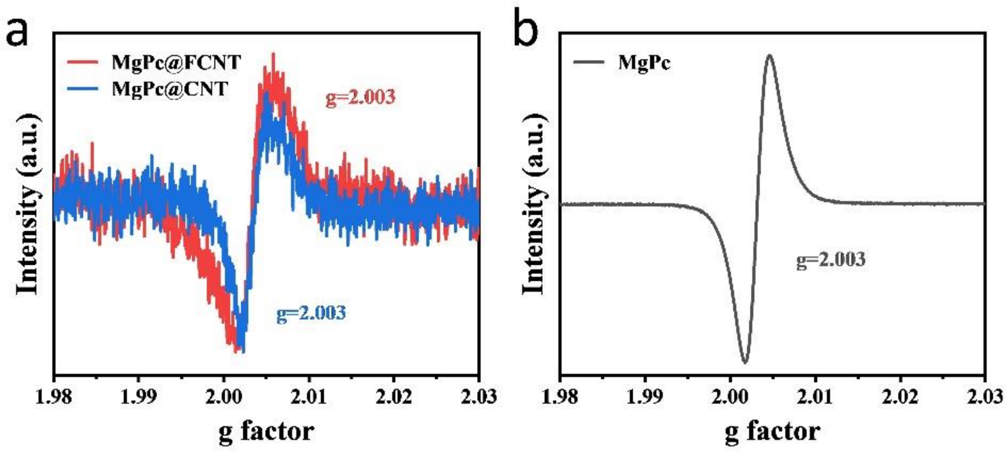

Fig. S11 The expanded peaks of EPR spectra for **a** MgPc@FCNT, MgPc@CNT and **b** MgPc

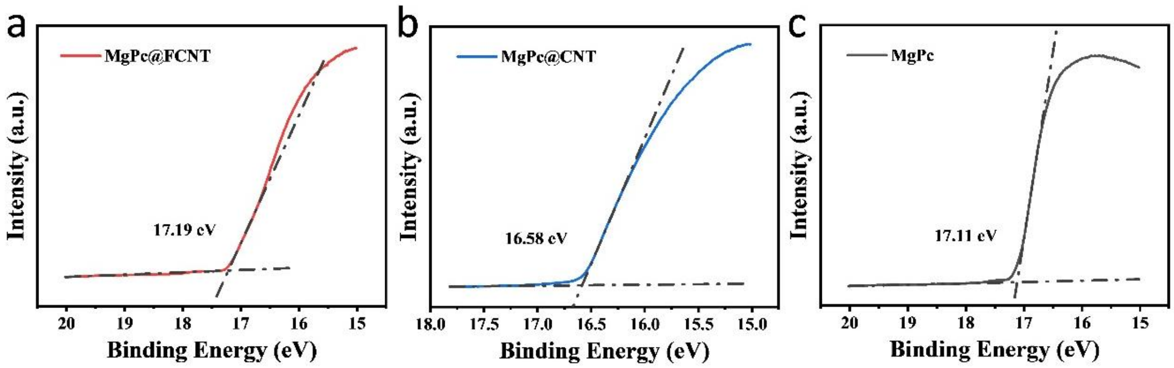

Fig. S12 Inelastic electron cutoff edge energy (E_{cutoff}) of **a** MgPc@FCNT, **b** MgPc@CNT and **c** MgPc calculated by UPS

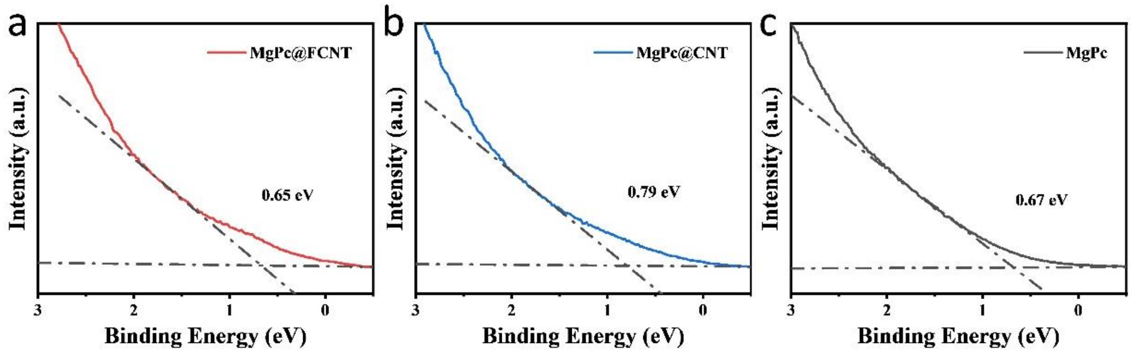

Fig. S13 HOMO energy of **a** MgPc@FCNT, **b** MgPc@CNT and **c** MgPc calculated by UPS

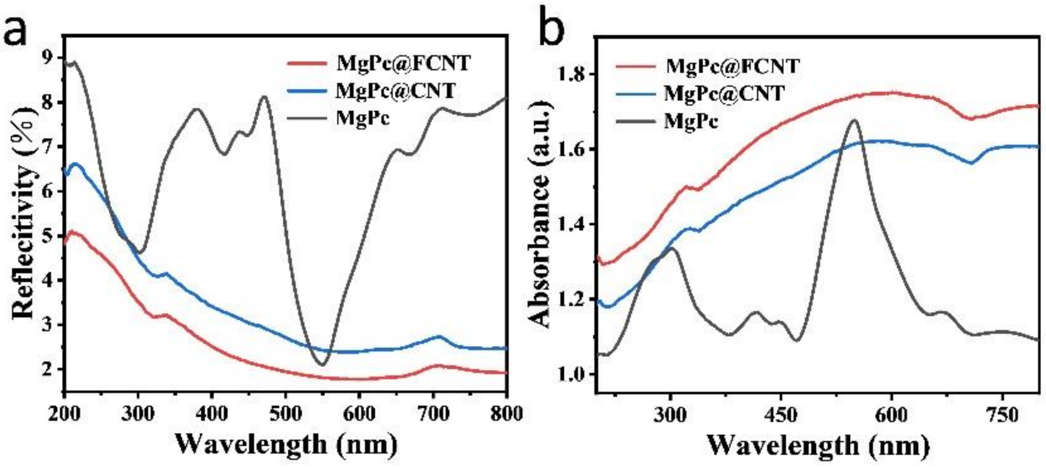

Fig. S14 UV-vis spectra of MgPc@FCNT, MgPc@CNT and MgPc. **a** Reflection spectrum, **b** absorption spectrum

Figure S13a shows the relationship between reflectivity and wavelength, and the relationship between absorbance and reflectance is as follows:

$$\alpha = -\log(R) \qquad \text{(S1)}$$

The relationship between absorbance and wavelength was obtained by replacing reflectance with absorbance using Equation 1.1, as shown in Fig. 13b. According to the Kubelka Munk equation:

$$(\alpha h\nu)^{1/n} = A\,(h\nu - E_g) \qquad \text{(S2)}$$

Among them α is the absorbance, h is the Planck constant, ν Is the frequency of light, A is a physical quantity related to the material, E_g is the bandgap energy value, n is 1/2 for direct bandgap semiconductors, and 2 for indirect semiconductors. Fig. 2e is obtained after converting Fig. S13b into photon energy. In Fig. 2e, select the straight-line segment with the highest slope as the tangent, and the value at the intersection of the tangent and the x-axis is the bandgap width [S8].

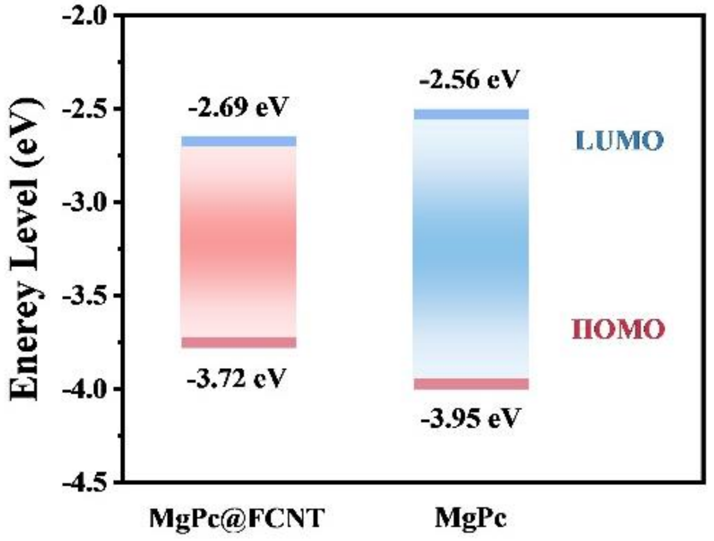

Fig. S15 HOMO/LUMO level alignment obtained through DFT calculations

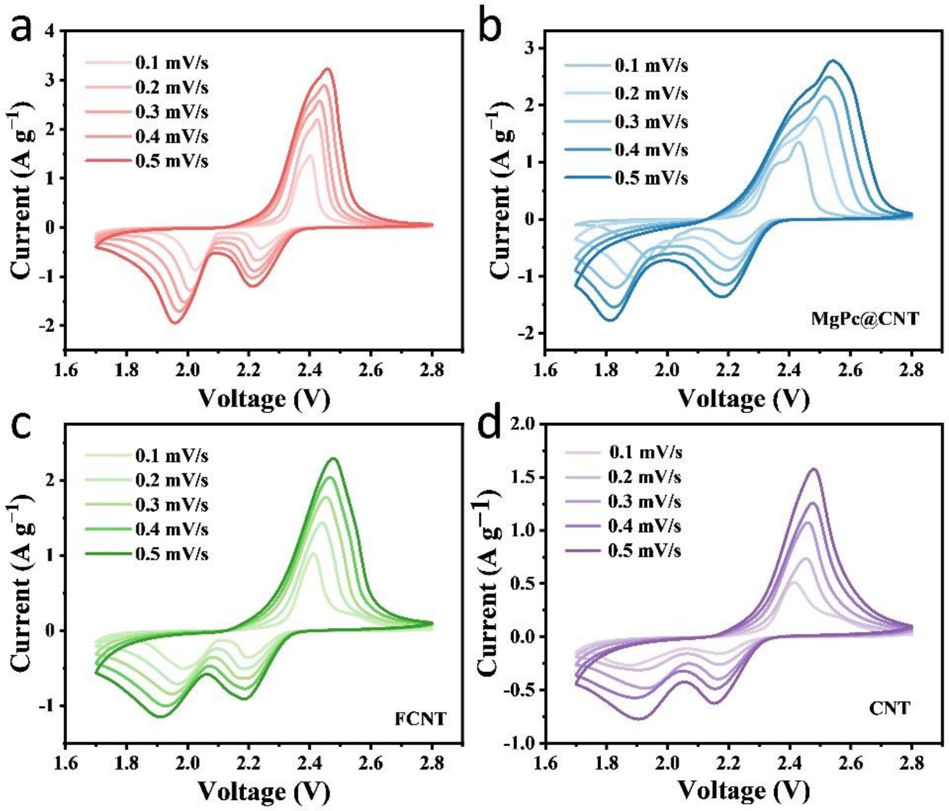

Fig. S16 CV profiles of **a** MgPc@FCNT, **b** MgPc@CNT, **c** FCNT and **d** CNT

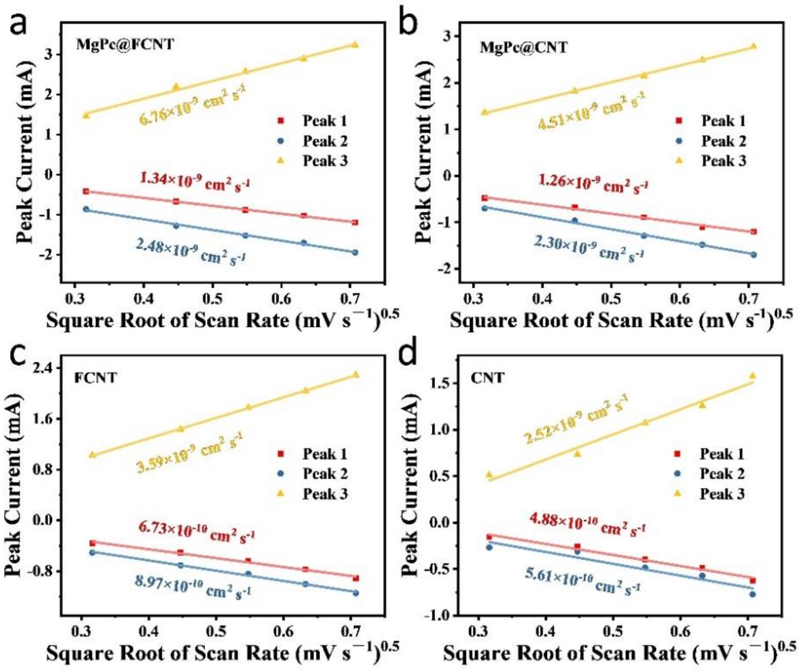

Fig. S17 Li-ion diffusion coefficient of **a** MgPc@FCNT, **b** MgPc@CNT, **c** FCNT and **d** CNT probed by the plots of CV peak currents vs. square root of the scan rates

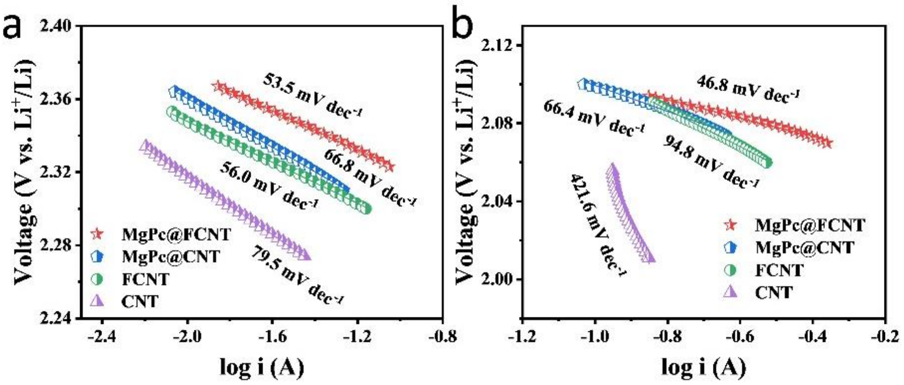

Fig. S18 Tafel slope of CV **a** peak 1 and **b** peak 2

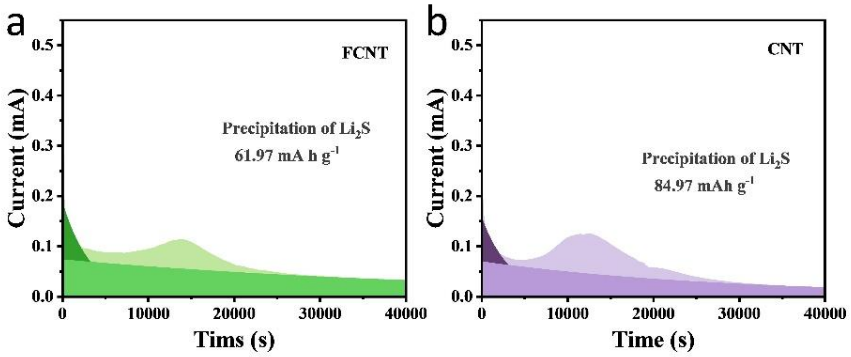

Fig. S19 The constant potential discharge curves of **a** FCNT and **b** CNT using Li_2S_6 solution at 2.05 V

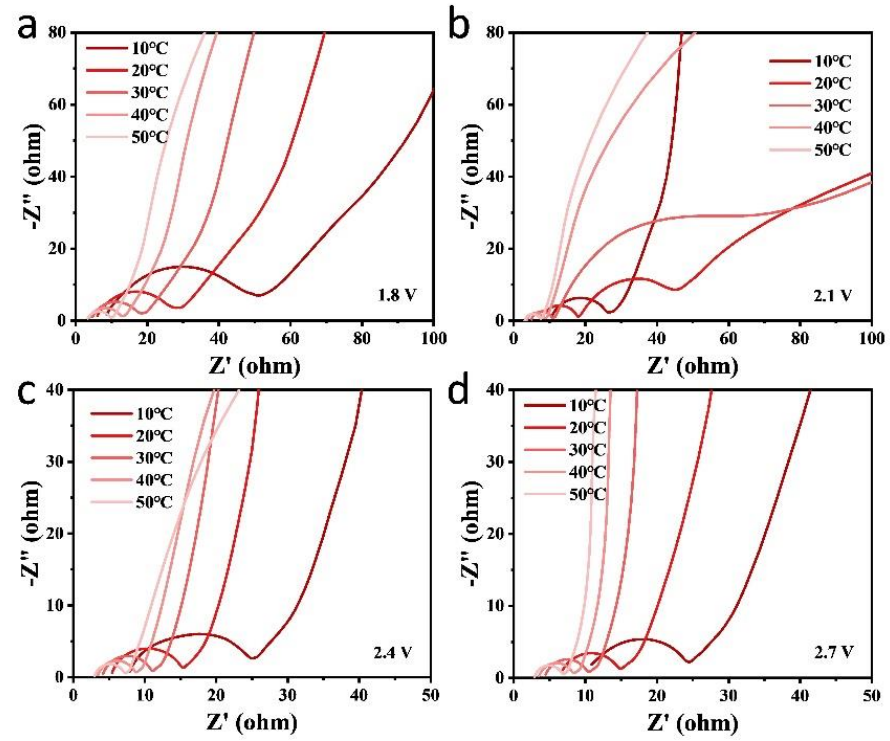

Fig. S20 Temperature-dependent EIS of MgPc@FCNT at 1.8, 2.1, 2.4 and 2.7 V

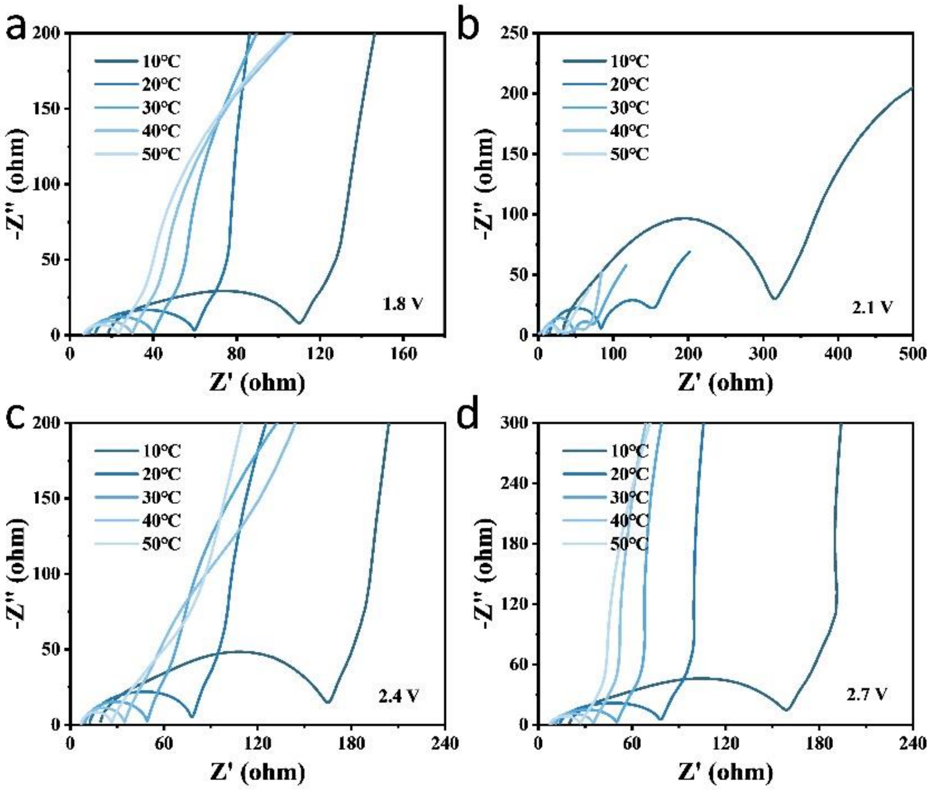

Fig. S21 Temperature-dependent EIS of MgPc@CNT at 1.8, 2.1, 2.4 and 2.7 V

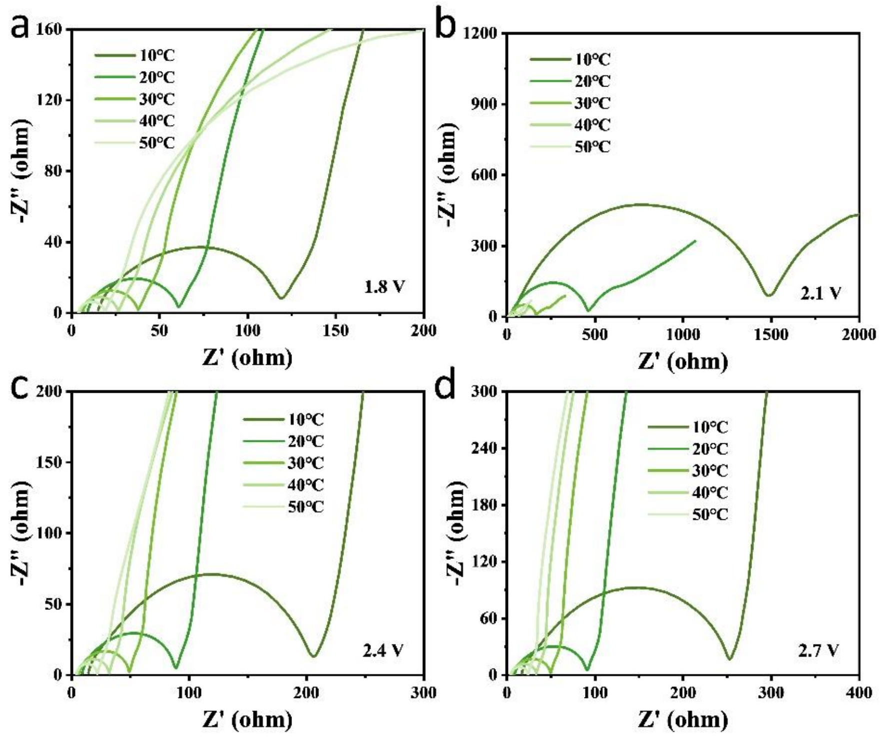

Fig. S22 Temperature-dependent EIS of FCNT at 1.8, 2.1, 2.4 and 2.7 V

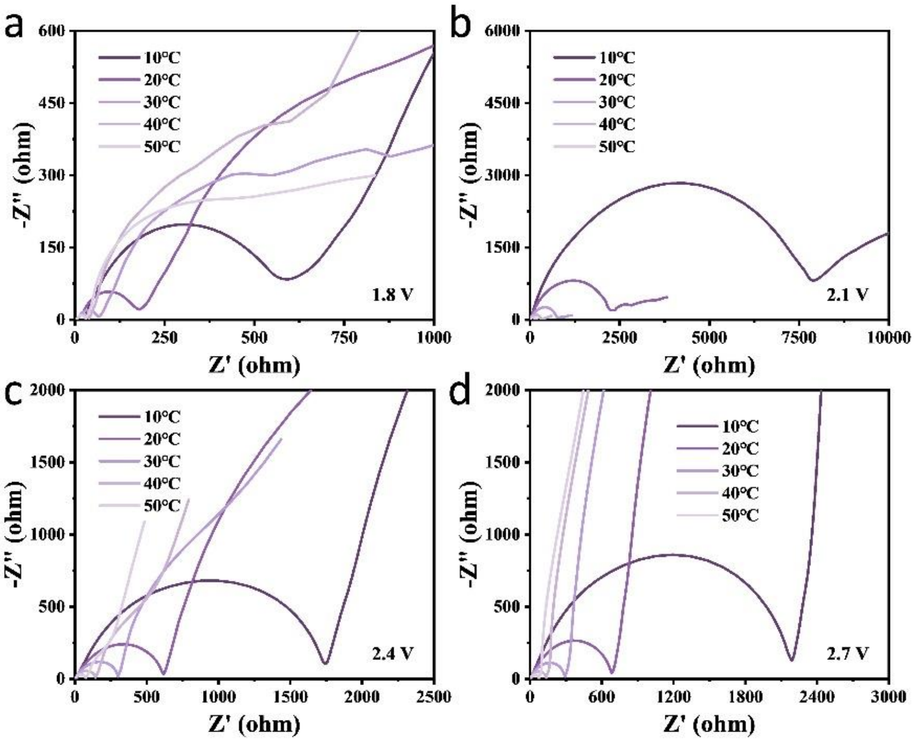

Fig. S23 Temperature-dependent EIS of CNT at 1.8, 2.1, 2.4 and 2.7 V

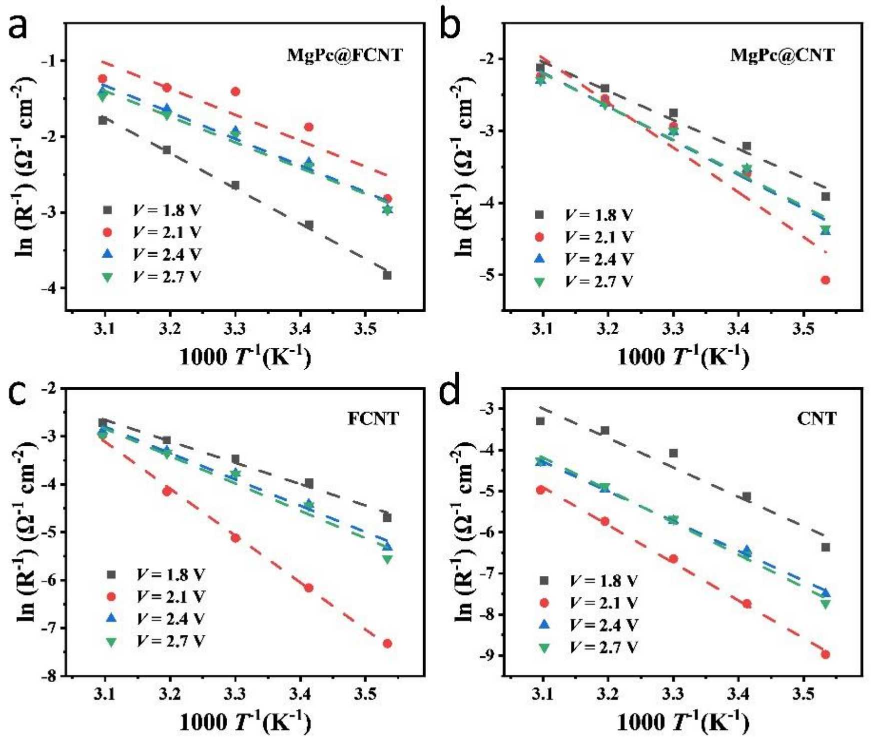

Fig. S24 Arrhenius plots of **a** MgPc@FCNT, **b** MgPc@CNT, **c** FCNT and **d** CNT batteries calculated based on the EIS curve

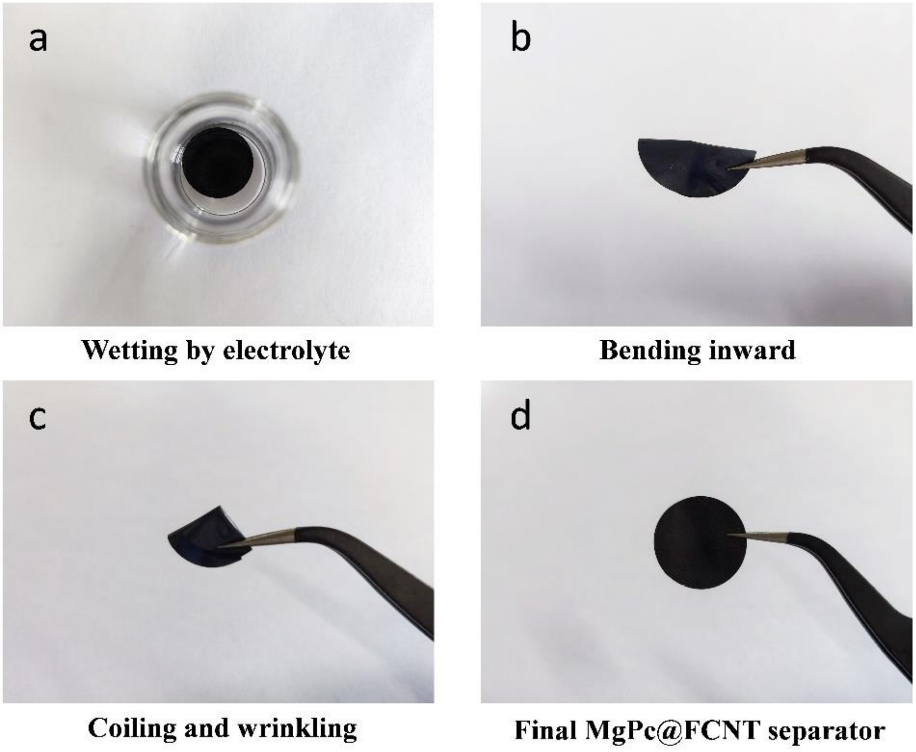

Fig. S25 Digital photos of the MgPc@FCNT modified separator under various mechanical stresses

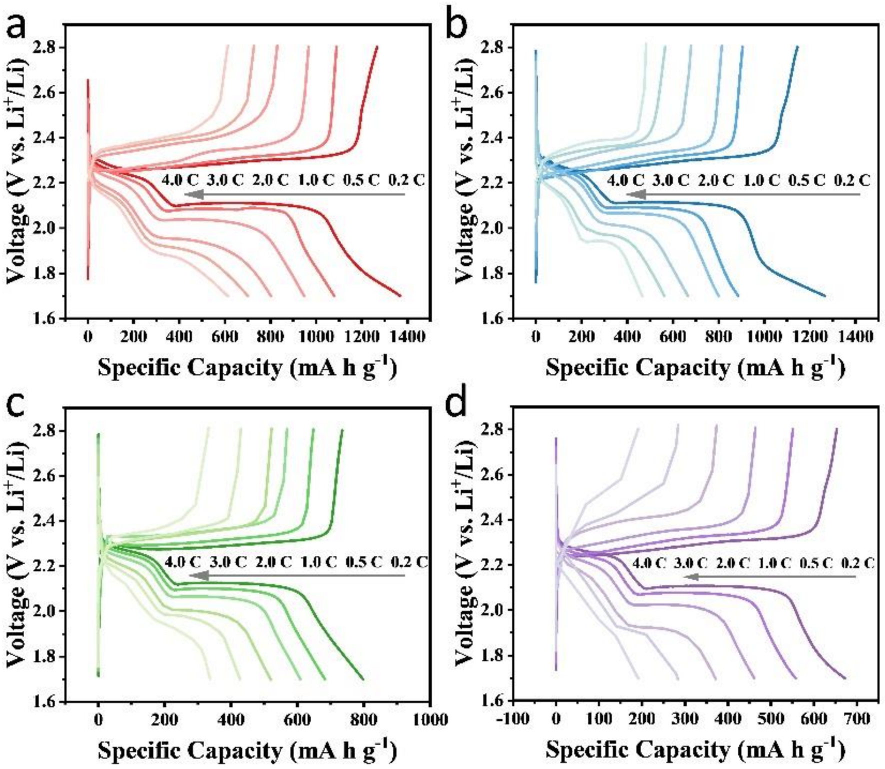

Fig. S26 The galvanostatic charge-discharge profiles of **a** MgPc@FCNT, **b** MgPc@CNT, **c** FCNT and **d** CNT at varied current densities

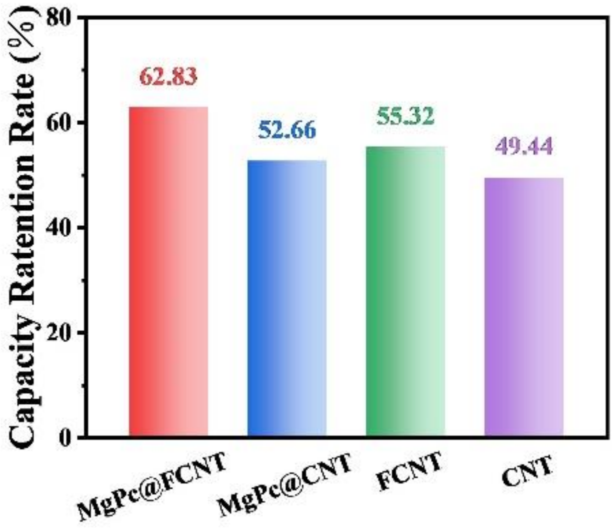

Fig. S27 Q2/Q1 at different rate of MgPc@FCNT, MgPc@CNT, FCNT and CNT

The *in-situ* XRD spectra were processed in air during the GCD processes for the MgPc@FCNT and MgPc@CNT. The voltage programming of the XRD patterns are shown in the left of Figs. 4d and S28.

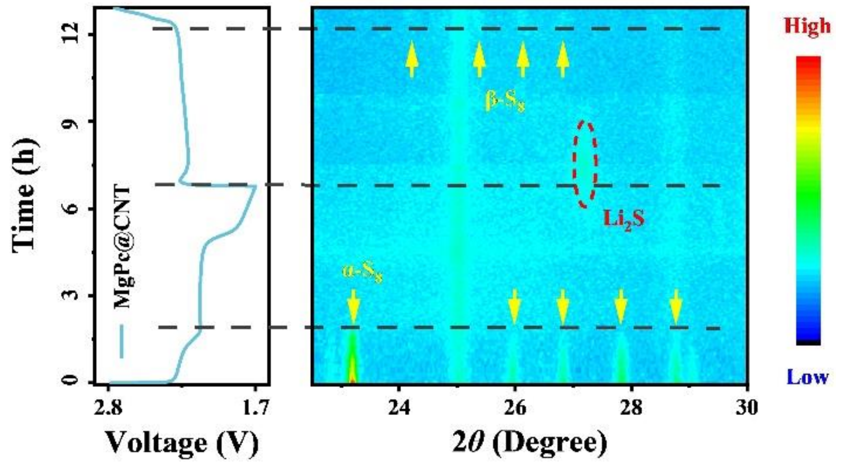

Fig. S28 2D pseudo-color in-situ XRD patterns of MgPc@CNT during the GCD processes

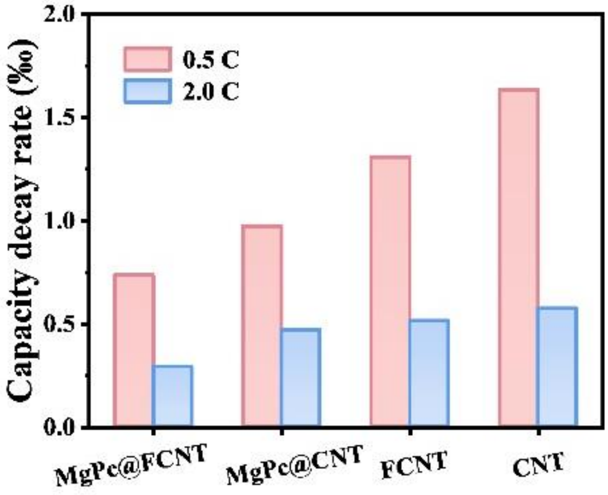

Fig. S29 The capacity decay rates at 0.5 and 2 C

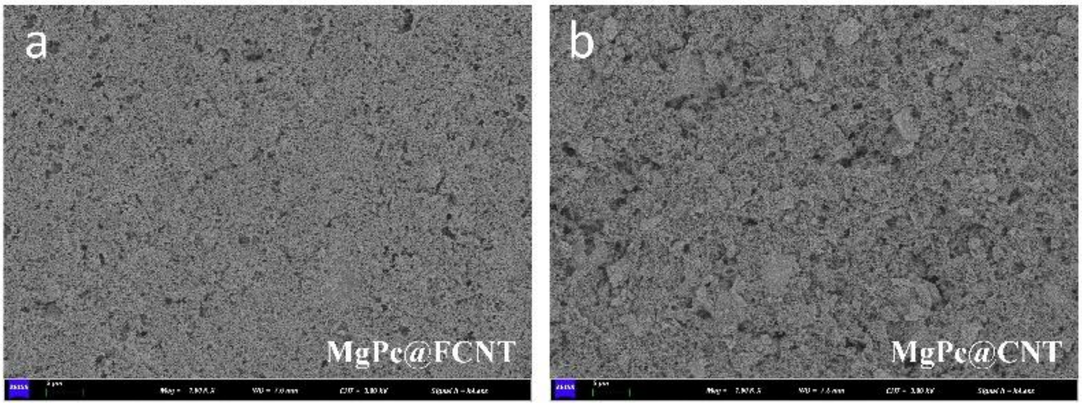

Fig. S30 SEM images of the cathode electrode after 100 cycles with a PP separator modified by **a** MgPc@FCNT and **b** MgPc@CNT

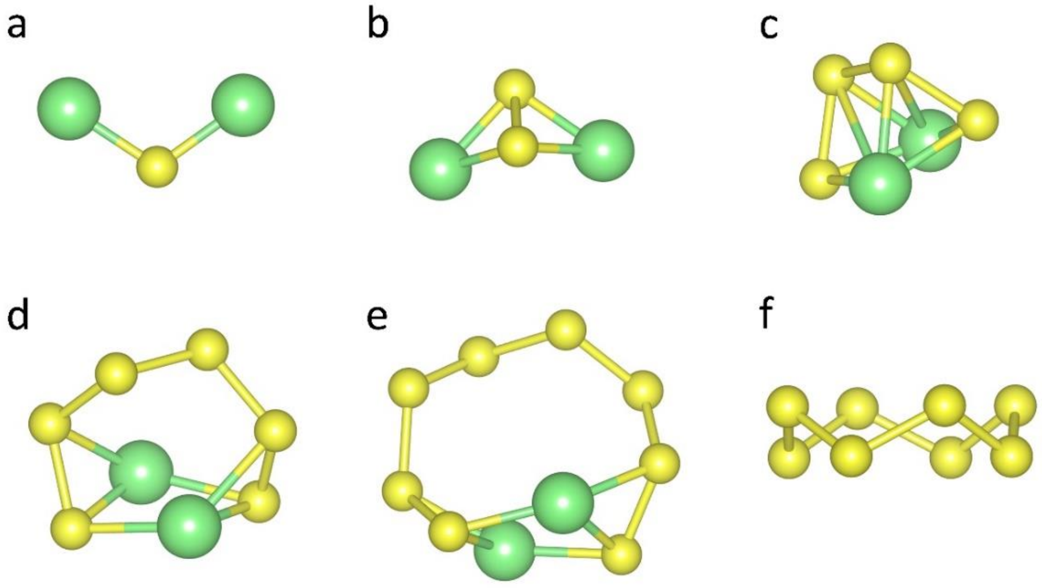

Fig. S31 Optimized Configurations of Li_2S_n (n=1, 2, 4, 6, 8) and isolated S_8 clusters. The Li and S atoms are green and yellow respectively

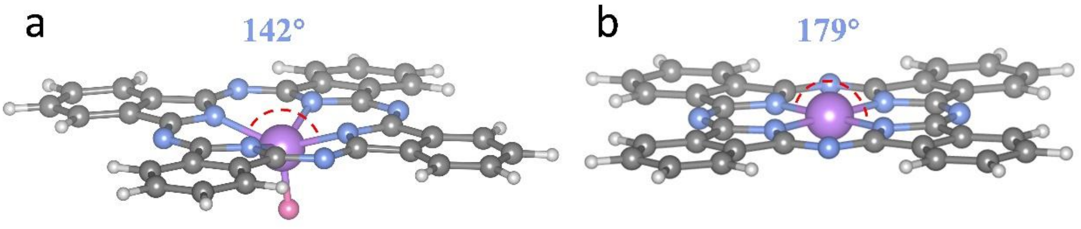

Fig. S32 Side views of **a** MgPc@FCNT and **b** MgPc@CNT. The H, C, N, Mg, and F are white, gray, blue, purple, and pink, respectively

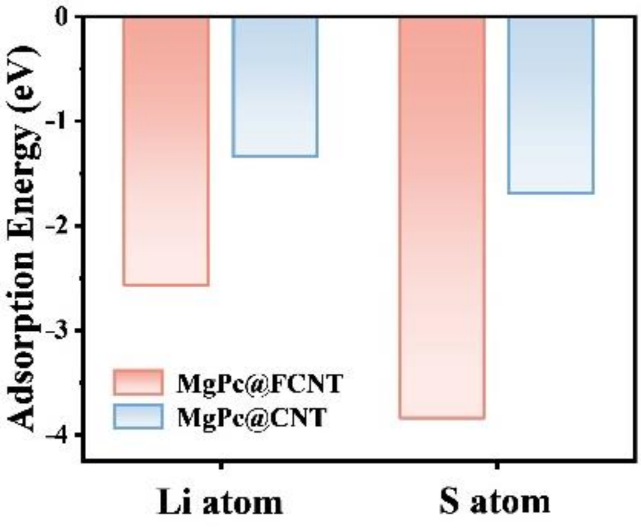

Fig. S33 Calculated atomic adsorption energy

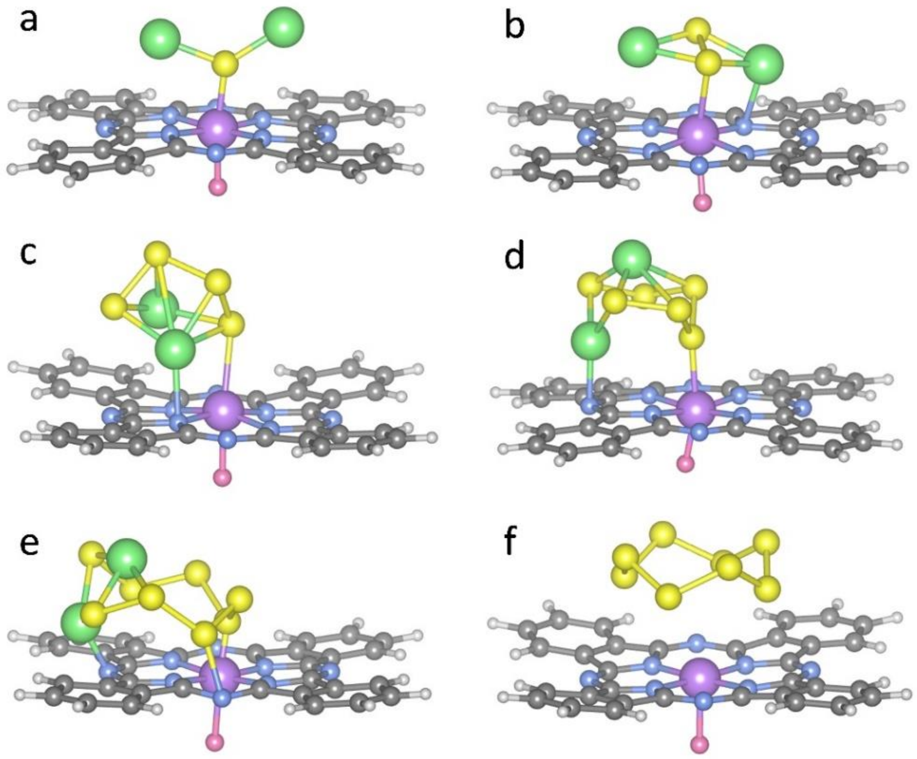

Fig. S34 Side views of the adsorption configurations of Li_2S_n (n = 1, 2, 4, 6, 8) and isolated S_8 clusters on MgPc@FCNT

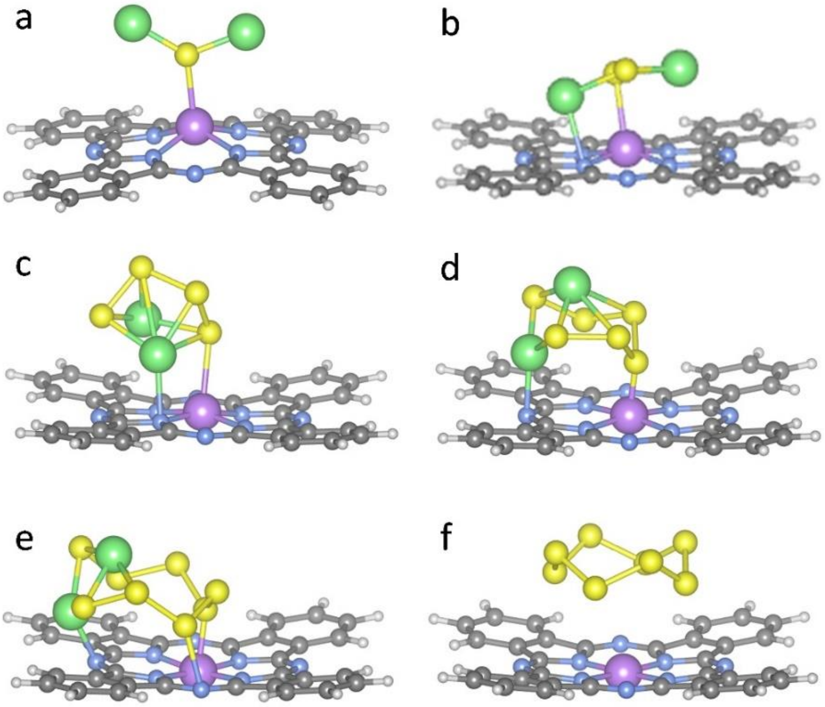

Fig. S35 Side views of the adsorption configurations of Li_2S_n (n = 1, 2, 4, 6, 8) and isolated S_8 clusters on MgPc@CNT

Table S1 The Mg contents in MgPc@FCNT as determined by ICP, XPS ang EDS mapping

Sample	ICP (wt%)	XPS (wt%)	EDS mapping (wt%)
**MgPc@FCNT**	0.36	0.73	0.57

Table S2 Vibration types of FT-IR peaks [S9]

Wavenumbers	Vibration types
**1330、1282、1165**	The stretching vibration of C-N bond
**1607**	The stretching vibration of C=N bond
**1078、1056、756、726**	The stretching vibration of phthalocyanine skeleton
**888**	Metal ligand vibration
**1482**	The stretching vibration of C=C bond

Table S3 The structural parameters at the Mg K-edge extracted from quantitative EXAFS curve-fittings

Sample	bond type	CN*	R (Å)	σ^2 (10^{-3}Å^2)**	R factor
Mg-SAC	Mg-N	3.0 ± 0.5	2.01 ± 0.01	5.4 ± 1.6	0.056
	Mg-F	2.1 ± 0.6	2.38 ± 0.02	5.4 ± 1.6	
MgPc	Mg-N	4	1.91 ± 0.03	11.4 ± 6.1	0.038
* CN: coordination number; S_0^2 was fixed from the fitting result of MgPc					
** σ^2: Debye−Waller factors					

Table S4 The bond length (Å) for Li_2S_n (n = 1, 2, 4, 6, 8) and isolated S_8 clusters on MgPc@FCNT and MgPc@CNT

	Adsorption	Li_2S	Li_2S_2	Li_2S_4	Li_2S_6	Li_2S_8	S_8
**MgPc@FCNT**	Mg–S bond	2.06	2.54	2.65	2.36	1.72	3.8
	Li–N bond		2.21	2.25	2.08	1.86	
**MgPc@CNT**	Mg–S bond	2.46	2.54	2.65	1.91	2.11	3.96
	Li–N bond		2.4	2.25	2.13	2.04	

Supplementary References

[S1] G. Kresse, J. Furthmüller, Efficient iterative schemes for*ab initio* total-energy calculations using a plane-wave basis set. Phys. Rev. B **54**, 11169–11186 (1996). https://doi.org/10.1103/physrevb.54.11169

[S2] G. Kresse, J. Hafner, *Ab initio* molecular-dynamics simulation of the liquid-metal–amorphous-semiconductor transition in germanium. Phys. Rev. B **49**, 14251–14269 (1994). https://doi.org/10.1103/physrevb.49.14251

[S3] P.E. Blöchl, Projector augmented-wave method. Phys. Rev. B **50**, 17953–17979 (1994). https://doi.org/10.1103/physrevb.50.17953

[S4] G. Kresse, D. Joubert, From ultrasoft pseudopotentials to the projector augmented-wave method. Phys. Rev. B **59**, 1758–1775 (1999). https://doi.org/10.1103/physrevb.59.1758

[S5] J.P. Perdew, K. Burke, M. Ernzerhof, Generalized gradient approximation made simple. Phys. Rev. Lett. **77**, 3865–3868 (1996). https://doi.org/10.1103/physrevlett.77.3865

[S6] J.A. White, D.M. Bird, Implementation of gradient-corrected exchange-correlation potentials in Car-Parrinello total-energy calculations. Phys. Rev. B **50**, 4954–4957 (1994). https://doi.org/10.1103/physrevb.50.4954

[S7] S. Froyen, Brillouin-zone integration by Fourier quadrature: Special points for superlattice and supercell calculations. Phys. Rev. B **39**, 3168–3172 (1989). https://doi.org/10.1103/physrevb.39.3168

[S8] Y. Xu, M. Di, Y. Wang, L. Fu, Y. Du et al., Structure, bandgap and photoluminescence of fluorinated reduced graphene oxide. Diam. Relat. Mater. **114**, 108342 (2021). https://doi.org/10.1016/j.diamond.2021.108342

[S9] Z. Liu, X. Zhang, Y. Zhang, J. Jiang, Theoretical investigation of the molecular, electronic structures and vibrational spectra of a series of first transition metal phthalocyanines. Spectrochim. Acta, Part A **67**, 1232–1246 (2007). https://doi.org/10.1016/j.saa.2006.10.013
